# A Comprehensive Review of Computational and Experimental Studies on Skin Mechanics and Meshing: Discrepancies, Challenges, and Optimization Strategies

**DOI:** 10.3390/biomimetics11010004

**Published:** 2025-12-22

**Authors:** Masoumeh Razaghi Pey Ghaleh, Douglas Marques, Denis O’Mahoney

**Affiliations:** 1 Department of Mechanical and Industrial Engineering, Atlantic Technological University, H91 T8NW Galway, Ireland; denis.omahoney@atu.ie; 2 Centre for Mathematical Modelling and Intelligent Systems for Health and Environment (MISHE), Atlantic Technological University, F91 YW50 Sligo, Ireland; 3 Department of Mechanical and Manufacturing Engineering, Atlantic Technological University, Ash Lane, F91 YW50 Sligo, Ireland; douglas.marques@atu.ie

**Keywords:** skin, constitutive models, skin meshing, severe burn treatment techniques, auxetic skin meshing pattern

## Abstract

Skin meshing is widely used to treat extensive burn injuries due to its cost-efficiency and capacity to cover large wound areas. As biomimetics focuses on deriving engineering principles from biological structure–function relationships, this review examines how to optimize skin-meshing expansion and investigates factors contributing to reported discrepancies between clinical and manufacturer-reported expansion ratios. The biology and mechanical behavior of skin layer are discussed, emphasizing the anisotropic properties govern by collagen fiber orientation associated with Langer’s lines in the dermis. The epidermis and hypodermis show isotropic properties and therefore have minimal influence on load-bearing capacity. Surveying 111 studies, the review evaluates which constitutive equations employed for skin modelling is suitable to replicate mechanical behavior of skin meshing undergoing large expansion. Elastic models fail to capture large expansion ratios. Viscoelastic and QLV are excluded due to negligible sliding of collagen fibers at slow strain rates and limited importance of hysteresis. Consequently, hyperelastic models are recognized as more suitable for predicting large deformations. Among these, the structural GOH model, which represents fiber dispersion through a probability-density function, demonstrates strong agreement with experimental data using few parameters; its damage extensions improve prediction of mesh tearing. Additionally, emerging auxetic mesh geometries with negative Poisson ratios are examined, highlighting their potential to achieve greater expansion when combined with suitable structural anisotropic constitutive models, e.g., GOH.

## 1. Introduction

A burn is a skin injury primarily caused by hot liquids, hot surfaces, flames, radiation, electricity, friction, and chemicals [1]. In 2011, burn injuries ranked fourth, surpassing tuberculosis and HIV, and are considered major contributors to the loss of disability-adjusted life-years (DALYs), a metric used to assess overall disease burden [1,2]. In October 2023, the World Health Organization (WHO) reported 180,000 burn-related deaths globally, with an estimated average cost of burn treatment of $88,218 per patient [1]. However, this does not include the additional expenses associated with burns, such as extended care for resulting disfigurement and treatment for the emotional trauma experienced by patients [1]. The extent of burns is measured by the total body surface area (TBSA) affected. Burns that cover more than 30% TBSA are considered severe and require medical treatment, such as skin grafting [2]. Skin grafting techniques include traditional sheet grafts [3], skin meshing [4,5], and the more advanced meek technique [6]. Sheet grafts require large donor areas and are prone to inflation [3], while the meek technique is costly and often yields less favorable cosmetic results [7,8]. In contrast, skin meshing remains the most practical option, offering an affordable approach capable of covering large wound areas [9,10,11]. In skin meshing, a skin mesher creates multiple slits on the skin’s surface to increase the graft’s meshing ratio or enhance skin expansion. The meshing ratio is the area of the skin after stretching divided by its original area [12]. However, a discrepancy exists between the expansion of meshing ratios claimed by manufacturers and those achieved in practice [13,14,15]. 

To understand this discrepancy between theoretical and clinical expansion, it is essential to examine how skin behaves mechanically during large expansion. Skin deformation is governed primarily by the dermis, where the orientation and dispersion of collagen fibers, which are associated with Langer’s lines as the natural tension of skin, produce anisotropic mechanical responses [16,17]. The epidermis and hypodermis, being largely isotropic, contribute minimally to load-bearing [18]. Yet, despite the crucial role of dermal anisotropy, there is a dearth of studies on whether Langer line directions are assessed relative to stretching directions, why practical expansion is lower than expected, and how meshing outcomes can be optimized [19].

In the literature, Computational modelling using the Finite Element Method (FEM) provides a framework to explore the mechanical behavior of skin models by fitting to experimental data. Many existing FEM studies adopt only a simple single layer of isotropic hyperelastic models, such as the Yeoh or Mooney–Rivlin models, for skin simulation, which cannot capture the anisotropic reinforcement provided by collagen fibers or the large deformation [12,20,21,22]. However, in this paper, more advanced constitutive equations, such as structural models that account for underlying collagen fiber dispersion and orientations, are investigated. The best constitutive model for large deformation of skin meshing was selected based on two criteria: the minimum number of model parameters required to fit the experimental data, and a stronger physical connection to the structure of skin. Among all suggested skin models, Gasser Ogden Holzapfel (GOH), with an accuracy of fitting of approximation of 99%, best fits the structure of skin [23]. This model embeds collagen fibers and is governed by the Von Mises distribution, which replicates the dispersion of fiber distribution [24]. Furthermore, the GOH- coupled with a damage model extends this capability by representing both large deformation and failure, offering more realistic simulations of mesh tearing [25].

These models only replicate the best-fit mechanical behavior of the skin meshing model under large deformation. However, to optimize the skin meshing model, recent work has shown that incision orientation, such as auxetic models and stretch direction, strongly influences the meshing ratio [15,23]. Auxetic patterns with negative Poisson ratios exhibit greater biaxial expansion than traditional slit designs. Combining appropriate constitutive models with optimized mesh geometries offers a promising pathway to improve graft outcomes [26].

**Figure 1:**
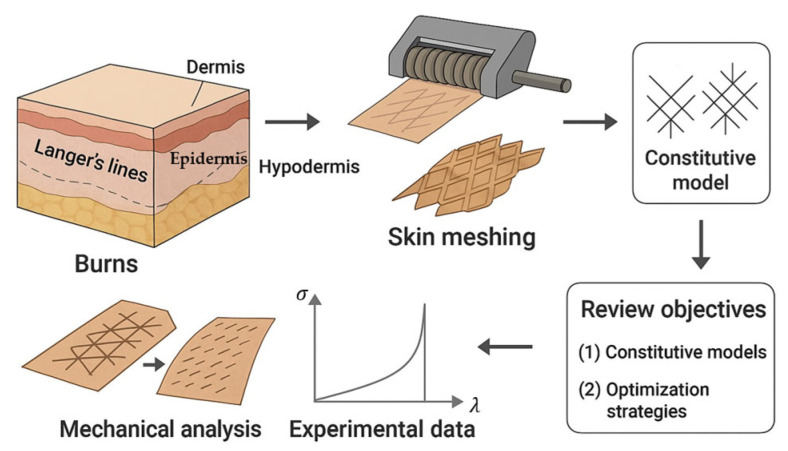
Schematic overview of the clinical and mechanical context of skin meshing. Severe burns often require grafting, and skin meshing expands harvested skin to increase wound coverage. The mechanical response during meshing is governed primarily by the dermis and its collagen alignment along Langer’s lines. Experimental stress–stretch data (σ−λ) and computational modelling are used to evaluate suitable constitutive models and meshing strategies. The review focuses on identifying an appropriate anisotropic material model and on exploring geometric optimization approaches to improve expansion outcomes.

Consequently, this review aims to evaluate: (I) the existing constitutive models that capture skin behavior and select the best structural model with a minimal number of fitting parameters to fit experimental data and replicate the anisotropic, nonlinear skin model with embedded collagen fibers in the dermis layer. (II) identifies which constitutive models merged with auxetic geometries, considering Langer’s line, could optimize the meshing ratios. The schematic, in Figure 1, also summarizes the motivation and framework of the review. This focus positions the work within biomimetics, as the constitutive modelling of skin meshing inherently relies on reproducing the dermis’s collagen-driven anisotropic mechanics.

A total of 111 studies published between 1973 and 2024 were selected from Elsevier, Springer, Taylor & Francis, MDPI, and PubMed. The search strategy, inclusion criteria, and review methodology are described in the following section.

## 2. Biology of Human Skin

**Figure 2:**
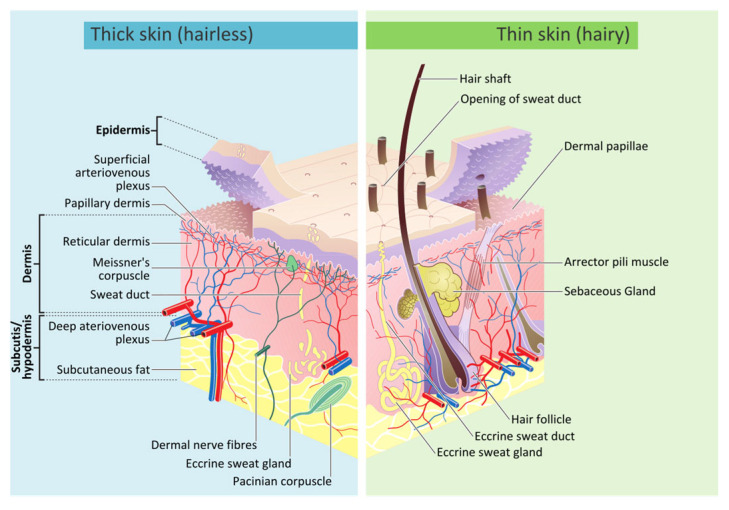
The structure of human skin (Adapted from [34]).

Skin serves as the outermost protective layer against external agents like heat, infections, and injuries. It also acts as an interface with the environment, containing various sensors, glands, channels, and pores that detect touch, temperature, and pain while regulating temperature and moisture [27]. Human skin consists of three main layers: epidermis, dermis, and hypodermis, as shown in Figure 2. The epidermis acts as a protective barrier against harmful external substances, and its thickness varies from 30 to 80 µm [17]. This layer constantly renews itself through stem cells in the basal layer, which differentiate into keratinocytes. Stem cells are basic cells that can turn into various types of cells. The basal layer is the deepest layer of the skin where these stem cells are located, and keratinocytes are skin cells that produce keratin, a protein that helps form the skin’s protective outer layer [28]. These keratinocytes migrate outward and transition from a columnar shape in the basal layer to a polygonal shape in the spinous layer [29]. Eventually, the keratinocytes differentiate into dead corneocytes, forming the stratum corneum, a tough keratinized layer that serves as a protective barrier against harmful substances [28,30]. The stratum corneum is stiff, incompressible, and isotropic, with its mechanical response largely governed by its water content [16]. Beneath the epidermis lies the dermis, with a thickness of 150 µm to 4 mm, rich in collagen and elastin fibers, which provide the strength and stretchability of the skin, respectively [17]. The semi-gel ground matrix is embedded in this layer as an amorphous mass of proteoglycans and water. The water molecules are not freely moving and are held together by hygroscopic hyaluronic acid, a constituent of proteoglycans [31]. Proteoglycans are large molecules in the body that consist of a protein core with attached glycosaminoglycan chains, playing crucial roles in maintaining the structure and hydration of tissues.

The dermis dominates the skin’s mechanical properties due to its dense arrangement of wavy collagen fibers (~60–80% of dry tissue weight) and elastin fibers (~1–4%) [16,31]. Elastin fibers and the ground matrix significantly influence the skin’s mechanical response only at low strains [16]. The hypodermis, which is composed of fats that anchor the skin to underlying muscles and bones, provides thermal insulation, energy storage, and shock absorption [32,33].

Understanding the layered structure and mechanical composition of skin provides the basis for interpreting classical biomechanical observations, such as Langer’s findings on natural skin tension.

One of the earliest biomechanical studies of skin dates to 1880, when Langer observed that circular incisions in human cadavers formed elliptical wounds [3,35,36]. In fact, the formation of the incision depends on its orientation, revealing the nonuniform natural tension in the skin [16,37]. Histological studies link skin’s natural tension, known as Langer’s line, to the arrangement of collagen fibers. 

**Figure 3:**
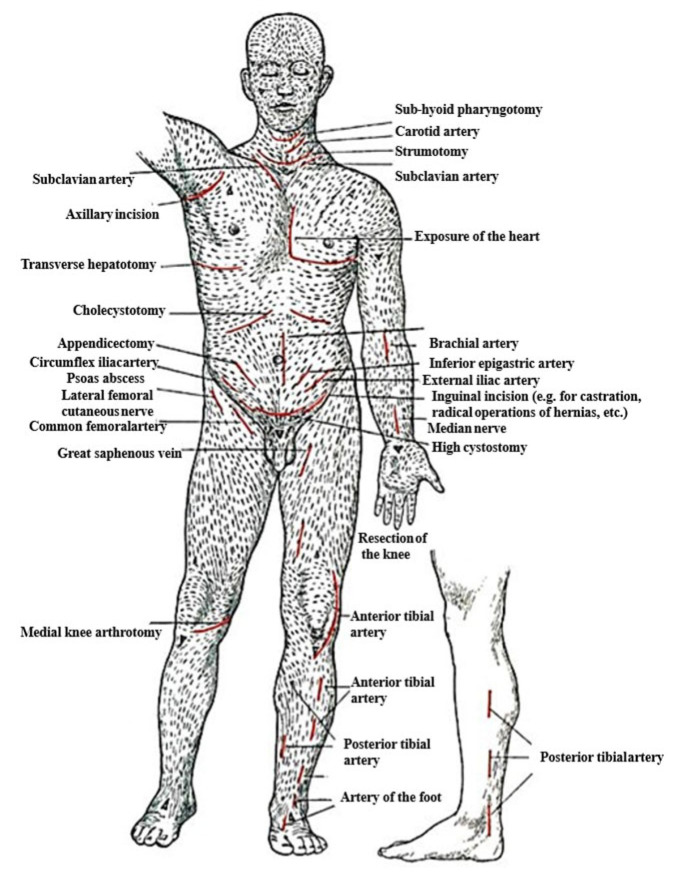
Langer’s line orientation across the human body (Adapted [42]).

**Figure 4:**
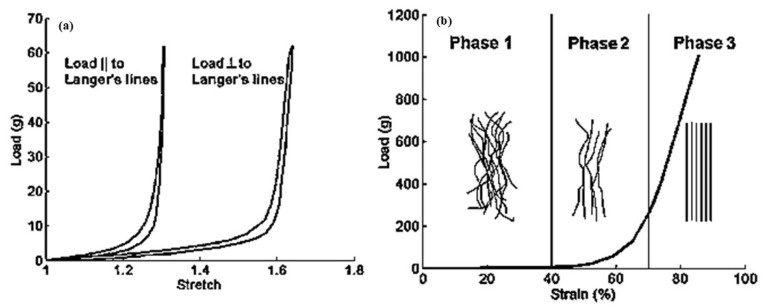
(**a**) In vitro, rabbit abdominal skin was subjected to biaxial stretch tests. The skin exhibited distinct anisotropic, time-dependent (viscoelastic) responses depending on the orientation of the applied load relative to Langer’s lines. Moreover, a notable degree of hysteresis was observed during the loading and unloading phases (Replotted from [38]). (**b**) The nonlinear load-strain response observed during an in vitro uniaxial test conducted on human abdominal skin. Initially, collagen fibers straighten without supporting any load. Next, some fibers are entirely straight and begin to bear the load. Finally, most of the fibers are straightened and carry the full load (Replotted from [43]).

Langer’s lines, as shown in Figure 3, are associated with skin anisotropy, a key factor indicating that the skin’s mechanical response varies with the direction of the applied load relative to these lines [37]. This effect is shown in Figure 4a, where in vitro biaxial stretching of rabbit skin demonstrated distinct anisotropic behavior depending on the loading direction. The mechanical ultimate tensile test exhibited that the skin is stiffer when stretched along these lines [38,39]. Therefore, the maximum stress is higher when the skin is stretched parallel to these lines than when stretched perpendicular to them.

Moreover, tensile tests on both human and rabbit skin exhibited a nonlinear, anisotropic, viscoelastic stress-strain response, as shown in Figure 4a,b, and the skin was nearly incompressible [38,40,41]. These findings highlight that the mechanical response of the skin depends on both the direction and magnitude of applied forces, which is primarily governed by the distribution and orientation of collagen and elastin fibers within the dermis [16].

The fiber structure in the dermis layer is quite complex in each species. In 2015, Sherman et al. [44] showed that the structural organization of collagen fibers in rabbit skin begins with polypeptide chains forming a triple-helix structure. Peptide bonds are chemical links between amino acids in proteins, formed when the carboxyl group (a carbon atom double-bonded to an oxygen atom and bonded to a hydroxyl group (-OH)) of one amino acid reacts with the amino group of another, releasing water and creating a covalent bond.

Tropocollagen molecules are the basic building blocks of collagen fibers, which consist of three polypeptide chains twisted into a triple helix, known as a superhelix. These molecules are approximately 300 nm long and 1.5 nm in diameter. These molecules organize into collagen fibrils with a characteristic d-period (67 nm) known as a staggered pattern, defining the structure of collagen. The fibrils, measuring ~50–300 nm in diameter, align parallelly to form fibers. These fibers, in turn, bundle together, forming structures with diameters of ~2–7 µm. The complexity of this arrangement at the fiber bundle level is not yet fully understood [37]. However, using an Aperio ScanScope XT scanner for human skin imaging and transmission electron microscopy for pig skin, the mean preferred orientation of collagen fibers, referring to the dominant directional alignment within the tissue, was reported as ±41° and ±45°, respectively [23,45].

## 3. Burn Treatment Techniques for Human Skin

The literature documents skin grafting [38] as a severe burn treatment technique, including traditional sheet grafts [36], skin meshing [38], and more advanced techniques, known as the meek technique [6,40]. In this section, the strengths and limitations of each technique are elaborated, and it is concluded which technique yields acceptable aesthetic outcomes while being economically suitable.

### 3.1. Skin Grafting

Skin grafting is a procedure in which skin is removed from a donor site and transplanted to a wound site. Skin grafting is primarily categorized into Full-Thickness Skin Grafts (FTSG) and Split-Thickness Skin Grafts (STSG) [46]. In STSGs, the epidermis and part of the dermis are transplanted into the wound part, whereas in FTSGs, the epidermis and the entire dermis are transplanted into the wound part. The STSGs expedite the rehabilitation of the wound part due to the rapid self-healing of the donor site [3,47]. Conversely, FTSGs initially take longer for blood vessels to connect to the wound site, delaying early graft integration and healing. However, once revascularization occurs, where new blood vessels form to restore blood supply, the thicker dermis supports better blood flow, improving graft survival. FTSGs result in thicker, more resilient skin, which enhances cosmetic outcomes, especially in delicate areas like the face and palms, where durability is crucial [48]. Despite these benefits, FTSGs are typically reserved for late-stage reconstruction in sensitive regions due to a shortage of donor sites and morbidity caused by the harvesting of thicker donor skin. When selecting donor sites for skin grafting, factors such as skin quality, color, texture, UV, thickness, convenience, size, contractility, and scarring must be considered. Table 1 compares STSG and FTSG, highlighting their primary differences [48].

**Table 1:** Comparison of STSG and FTSG techniques (Adapted [3,35,36]).

	Split-Thickness Skin Graft	Full-Thickness Skin Graft
Composition	Epidermis + part of the dermis	Epidermis + entire dermis
Graft survival	Higher initial graft survival rate due to quicker revascularization	Lower initial graft survival rate due to more complex revascularization
Resistance to trauma Cosmetic appearance	Less resistant Poor cosmetic appearance owing to poor color and texture match. Does not prevent contraction.	More resistant Superior cosmetic appearance. It is thicker, preventing wound contraction or distortion.
Indications	Temporarily or permanently after removal of skin cancer with a high chance of recurrence. If a flap is not available in areas with limited vascular supply.	When aesthetic outcome is essential (e.g., facial defects).
Common uses	Chronic lower-leg ulcers (e.g., venous, irradiated tissues; exposed periosteum, cartilage, or tendon) Surgically induced significant defects (e.g., birthmarks, nevi)	Facial defects: nasal tip, dorsum, ala or side wall, lower eyelid, ear
Donor site tissue	Anteromedial thigh, buttock, abdomen, inner or outer aspect of the arm, inner forearm.	Donor sites with a similar color/texture to the defect (e.g., preauricular, postauricular, supraclavicular areas).
Disadvantages	Poor cosmetic appearance, higher chance of contraction, limitations in certain burn areas [49].	Greater risk of graft failure, prolonged healing time for the donor site, potential for hypertrophic scarring, and suitable for more minor burn treatments [48].

### 3.2. Sheet Graft

Sheet grafts are also a form of skin grafting that can be either STSGs or FTSGs. Sheet grafts are commonly employed for minor burns due to their superior aesthetic results. However, they require substantial donor sites, which restricts their use for extensive burns, and the lack of donor skin makes covering larger wounds difficult. Besides, the accumulation of blood or fluid beneath the graft can lead to clot formation, increasing the risk of graft detachment from the wound bed. This entrapment of fluids heightens the risk of infection and hinders the healing process [3].

Given the limitations of traditional skin grafting techniques, such as sheet grafts, the need to explore techniques for treating severe burns has become imperative. Skin meshing and meek micrografting techniques are the standard methods for surfacing extensive areas. These techniques tackle the limited availability of donor skin in severe burn treatment by establishing specific patterns on the skin surface [11,50].

### 3.3. Skin Meshing

**Figure 5:**
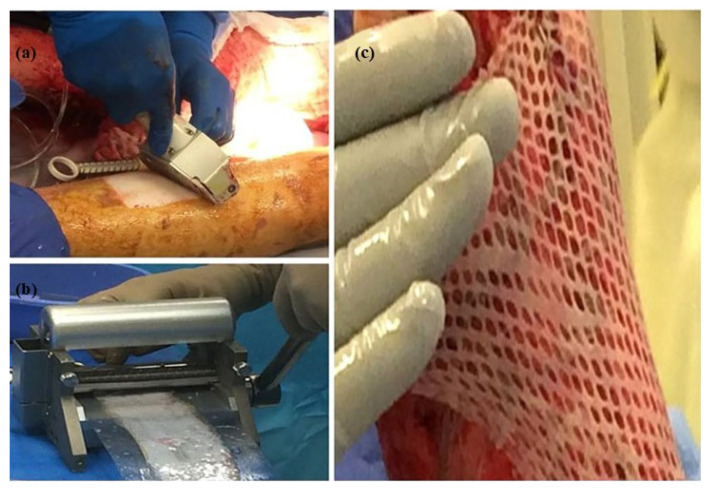
(**a**) Donor skin is removed by dermatome, (**b**) mesh is established using skin mesher, and (**c**) skin is expanded and covers a larger wound bed area (Adapted [55]).

Skin meshing, shown in Figure 5, served as the standard burned treatment technique due to its moderate cost and coverage of larger wound areas [11,51]. In this technique, surgeons harvest donor skin using a dermatome, a surgical instrument that precisely harvests thin layers of skin, and pass it through a device called a skin mesher to create multiple slits on the skin’s surface. The slit pattern, known as the mesh pattern, facilitates the expansion of the skin, effectively increasing its coverage area. The carrier’s meshing ratios determine the skin’s expansion, typically specified by manufacturers as 1.5:1, 2:1, or 3:1. For instance, a 3:1 ratio indicates that the skin can be expanded to three times its original area [15]. However, the skin meshers designed by manufacturers fail to achieve the claimed meshing ratios in practice [13,52,53]. The reason for the diverging expansion ratios has not been reported. However, the hypothesis is that due to the anisotropic properties of the skin, the mechanical behavior of skin depends on the direction in which the skin is stretched, correlating with the preferential orientation of the collagen fiber network [54]. Therefore, the orientation of stretching the skin meshing, which was overlooked by surgeons, could play a significant role in why claimed expansion ratios cannot be replicated in practice [20]. 

Section 5.1 examines the mechanical behavior of skin under large deformation using constitutive models. A suitable model enables realistic prediction of skin behavior and offers a cost-effective alternative to experimental methods. Additionally, evaluating different slit patterns, such as the auxetic patterns shown in Section 6, can help determine how to achieve an optimized skin meshing expansion.

### 3.4. Meek Technique

**Figure 6:**
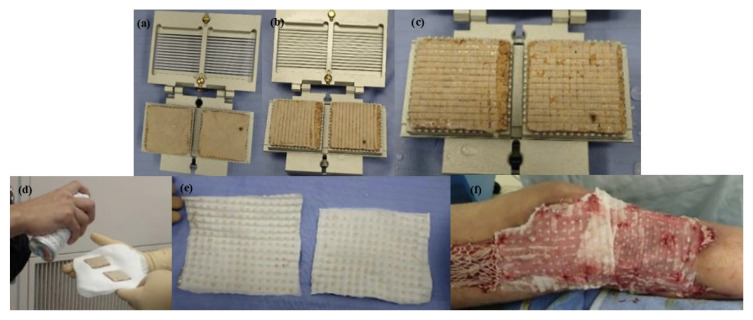
(**a**) The skin is placed on a moistened cork with the dermis facing down. (**b**) The skin is cut into 14 strips. (**c**) The skin is processed into meek micrografts by cutting it into tiny, square pieces. (**d**) An adhesive is sprayed on the epidermal surface of the micrografts. (**e**) The skin is biaxially expanded. (**f**) The expanded micrografts with gauze are fixed with a stapler on the burned site (Adapted [57]).

The meek technique, as shown in Figure 6, represents a significant advancement in skin grafting, particularly effective when donor skin availability is limited. The process begins with the skin being mounted onto a moistened cork carrier, with the epidermis facing down. Then, the mounter is inserted into a meek micrograft cutter, and the skin is cut into precise, tiny square pieces. Later, an adhesive spray is applied on the epidermis surface, and the micrograft skin is transferred to a gauze with the dermis facing down. Finally, the gauze is biaxially expanded and stapled onto the wound part. Despite its ability to achieve broad coverage of the wound area, this technique has challenges, such as the high cost of the meek micrograft cutter and a patchwork appearance at the wound site, which might be an aesthetic concern in patient care. Besides, the Meek technique requires a longer preparation time in parallel with wound preparation, such as negative-pressure wound therapy (NPWT), which involves applying a vacuum to the wound site to remove excess fluid, promote blood flow, and facilitate faster wound-healing. It also requires more precise placement of the micrograft skin on the mounter, gauze and wound area as compared to skin meshing [6,50,56].

Recent advancements have further evolved from the meek technique, introducing innovative methods such as minced micrograft and Xpansion^®^ micrografting. Minced micrografts involve dividing a small piece of skin into even smaller fragments to enhance coverage of the wound site. Xpansion^®^ micrografting expands the skin up to a hundred times its original area [58]. Despite the increased wound coverage offered by the meek technique compared to traditional sheet grafts and skin meshing, the meek technique was reported to have a significantly longer average surgery time of 181 min, compared to 80 min for skin meshing. This extended duration may increase the risk of complications such as infection, blood loss, and overall higher surgical costs [11]. Additionally, the meek device has much higher costs, which makes it less accessible [7,8].

Therefore, there is room for improvement in traditional burn treatment techniques, such as optimization of the performance of skin meshing, which are generally simpler, require less training, and are most cost-effective. Consequently, the following sections investigate clinical, experimental, and computational techniques for skin meshing to explore how the expansion ratio can be optimized by incorporating realistic skin properties into computer models.

## 4. Experimental and Clinical Studies on Skin Meshing

As the introduction outlines, more consistency is needed between the expansion of meshing ratios achieved by surgeons and those claimed by manufacturers. However, there is a dearth of experimental and clinical studies investigating why skin meshing fails to achieve the high expansion ratios claimed by manufacturers. Primarily, Vandeput et al. [19] categorized skin meshing instruments into three types: Mesh Dermatome type I, which uses a flat block roller with staggered cuts at a 15-degree angle; Mesh Dermatome II, featuring parallel rows of staggered cuts with a grooved carrier; and the Zimmer skin graft Mesher, which incorporates interchangeable cutters for various meshing ratios of 1.5:1, 2:1, 3:1, and 4:1 claimed by manufacturers with disposable plastic carriers with grooved pattern. The staggered cuts facilitate effective and uniform skin expansion by creating a honeycomb pattern that evenly distributes stress, reducing the risk of tearing. Vandeput highlighted that, in Zimmer skin grafting, the blades are designed to cut across the ridges into the skin while skipping the grooves and modifying the angle between the grooves. Adjusting the angle between the blades and grooves allows for a higher length cut of the skin sample, which enhances the meshing ratios. Furthermore, the grooves on the carrier control the interval space between slit cuts. The mathematical calculation of the skin mesher, which was used as a benchmark for fabricating the skin mesher, even exceeded the claimed expansion ratios by manufacturers precisely for meshing ratios of 1.5:1 and 2:1, whereas, in clinical settings, surgeons often observed immediate skin shrinkage post-harvesting due to skin’s elastic response, which they believed is the reason behind the mismatch of claimed expansion ratios by manufacturers and the clinical cases [19].

Following Vandeput’s report [19], Peeters et al. conducted a clinical analysis using the Zimmer mesh graft to examine the expansion ratios of skin mesher, comparing meshing ratios of 1.5:1 and 3:1. The study found actual meshing ratios of 1.2:1 and 1.5:1, respectively, indicating deviations from expected outcomes. They also observed, consistent with previous findings, that post-harvested skin tends to shrink and contract as the skin’s fibers naturally try to return to their original state due to the skin’s elastic response [53]. Additionally, Ratner [59] emphasized skin’s incompressibility and elastic response, hindering higher expansion ratios. The incompressibility of skin is due to its high-water content and dense extracellular matrix, which restricts any significant volume change.

Henderson et al. [52] also revisited the study on the meshing ratios of 1.5:1 and 3:1 for porcine skin grafts using a Zimmer mesh graft, noting discrepancies between the expected outcomes and the actual meshing ratios of 1.36 and 1.80, respectively. The discrepancy was attributed to limitations of the meshing equipment, including the carrier and the skin’s complex mechanical properties. They also introduced the concept of “over-meshing,” which involved an initial meshing at a ratio of 1.5:1 followed by a subsequent re-meshing in the same or an orthogonal direction at a ratio of 3:1. Findings indicated that over-meshing along the same axis as the initial meshing preserved the structural integrity of SSGs, whereas re-meshing orthogonally (cross-meshing) led to fragmentation of the skin into tiny, unusable pieces. The study highlighted that primary meshing achieved varying levels of skin meshing ratios, up to a factor of 2.3, with over-meshing emerging as a viable method achieving significant expansions of skin meshing.

The literature appears to lack evidence that surgeons consider the orientation of Langer’s lines during skin meshing expansion, despite its potential influence on meshing ratios, as discussed in the next section.

## 5. Computational Models of Skin

This section outlines the constitutive models, which are mathematical equations representing the material section employed in Finite Element Analysis (FEA) for various skin modelling and biological tissue, including human or pig skin, which exhibit similar properties [60] to determine if they replicate the nonlinear, anisotropic, and viscoelastic mechanical behavior observed in experimental skin testing under different loading conditions. The constitutive models are categorized based on elastic, hyperelastic, viscoelastic, quasi-linear viscoelastic, and damage models. The limitations and strengths of each model are evaluated to identify a constitutive model that most accurately replicates the anisotropic, nonlinear behavior of skin under large deformations, suitable for skin meshing applications. The viscoelastic behavior observed at large strains and low stress levels is attributed to the molecular sliding of collagen fibers [61]. However, this sliding process is significantly slowed and rendered almost negligible at low stretch rates. Therefore, the viscoelastic contribution becomes less dominant under the low strain rates typically used in characterization tests and the large expansions relevant to skin meshing, where collagen recruitment and the onset of damage govern the mechanical response. Therefore, incorporating full viscoelastic formulations is not essential in meshing simulations and only increases the computational cost [62]. 

Furthermore, a key criterion reported in the literature for evaluating constitutive models is the incorporation of histological analysis of human skin, ensuring that the model’s parameters are directly linked to its underlying structure and are measurable in the laboratory [63]. Therefore, the most suitable constitutive model for simulating skin meshing is based on its ability to fit the mechanical response of experimental data with the least number of parameters and errors which capture the skin’s nonlinear and anisotropic behavior under large deformations and maintain a clear physical connection to the dermal microstructure, ensuring both accuracy and biological relevance. 

The simplest classical linear elastic constitutive model, Hooke’s law, failed to represent the skin’s mechanical behavior [64]. This model is based on constant parameters for the Elastic modulus and Poisson’s ratio, which describe only small, linear stress-strain deformation and do not account for the skin’s nonlinear, anisotropic, and viscoelastic properties. Therefore, the subsequent sections present an overview of employed hyperelastic, viscoelastic, quasi-linear viscoelastic, and damage-based models that have been employed to more accurately characterize biological tissue and skin mechanical modeling.

All the mathematical representations of the discussed model are listed in Supplementary Materials Table S1.

### 5.1. Hyperelastic Constitutive Models

Hyperelasticity is designed to model rubber-like materials, where the elastic deformation can be extremely large. The stress–strain relationship in this model is derived from a strain energy density function, which defines the amount of energy stored per unit volume as the material deforms. These models are typically calibrated by fitting experimental data, such as force–displacement and stress–stretch (or stress–strain) responses, through the optimization of material parameters [65]. This section categorizes the hyperelastic models into classical, statistical (PDF-based) fiber dispersion, and discrete fiber-based framework models. 

#### 5.1.1. Classical Hyperelastic Models

These models typically use strain energy functions without direct reference to underlying tissue microstructure. The earliest efforts date back to 1976, when Tong and Fung [66] developed a hyperelastic model to replicate the rabbit abdominal skin’s nonlinear, anisotropic mechanical behavior, comprising an exponential strain energy function with nine constant material parameters. While the Fung model effectively captured the nonlinear and anisotropic mechanical response of force versus stretch of experimental biaxial rabbit skin data, its performance varied across different biaxial loading and preconditioning, demonstrating outcomes that ranged from good to moderate and poor agreement with experimental data.

Subsequently, the Fung model was extended by Ateshian et al. [67] through a frame-invariant formulation implemented in the FEBio finite element analysis (FEA) open-source software. This development generalized the Fung model to full three-dimensional representations, moving beyond the limitations of two-dimensional orthotropic plane stress assumptions. The revised formulation includes eleven material parameters (listed in Supplementary Materials Table S1) and accommodates both constrained (e.g., incompressible) and unconstrained formulations, enhancing its applicability for soft tissue modelling within three-dimensional FEA frameworks.

Bellini et al. [68] evaluated the performance of Fung hyperelastic constitutive models with classical Neo-Hookean, Mooney–Rivlin models, by fitting them to equibiaxial test data obtained from the intestine of adult Yorkshire pigs, collected 22 ± 10 hours post-harvest. As a biological tissue, the intestine shares key mechanical characteristics with skin, including nonlinearity, and anisotropy. The Neo-Hookean model is a simplified version of the Mooney–Rivlin model containing one constant parameter and demonstrating isotropic, nonlinear properties, and is only effective for small deformations. Both Neo-Hookean and Mooney–Rivlin showed poor average nonlinear regression (R^2^ = 0.45) of the stress-strain mechanical response of experimental data in the jejunum region. In contrast, the Fung model achieved a superior fit in the jejunum with an average of R^2^ = 0.87 for both circumferential and longitudinal directions. Despite capturing nonlinearity and anisotropy, the Fung model with four parameters does not account for the skin’s underlying microstructure.

Along similar lines of comparison, Shergold et al. [69] also evaluated classical Ogden (*N* = 1) and Mooney–Rivlin hyperelastic constitutive models, incorporating two and one parameters, to fit the uniaxial tensile engineering stress versus stretch of a few hours post-slaughter pig rump skin in parallel and perpendicular to the spine stretch directions. Both models capture the isotropic and nonlinear behavior of skin. However, neither model provided a good fit in both the transverse and longitudinal stretch directions, as they neglect the anisotropic behavior of skin. The Mooney–Rivlin model fitted the experimental data better at low stretches, whereas the Ogden model fitted both small and large deformations.

Building on these early efforts, the Ogden model was applied to in vivo experimental data by Flynn et al. [70] to improve accuracy through higher-order formulations and more representative experimental setups. When material parameters with *N* = 1 were used, an average fitting error of 30.3% was reported. By increasing the model complexity to *N* = 2, the average error was reduced to 15.8% for the anterior forearm and 19.2% for the posterior upper arm. These results were obtained by fitting in vivo force–displacement data, which had been acquired from the anterior forearm using a force-sensitive micro-robot.

In addition, attempts have also been made to integrate the underlying tissue structure into the classical constitutive models. For instance, Manschot and Brakke [71] developed a hyperelastic nonlinear constitutive model inspired by the viscoelastic Lanir’s equation [72], assuming perfectly aligned collagen fibers parallel to the epidermis with sinusoidal undulations, excluding its viscoelastic term. The fitted model to in vivo uniaxial data from human calf skin achieved a correlation coefficient of 0.998. However, the limitation of this model lies in its assumption of perfectly parallel collagen fiber alignment, which does not fully reflect the anisotropic behavior of the dermis layer.

#### 5.1.2. Fiber Distributed-Based Hyperelastic Models 

To account for the dispersed nature of collagen fiber orientations in biological soft tissues, constitutive models often incorporate probability density functions (PDFs), enabling a statistical representation of fiber architecture. This approach provides a means to capture the inherent anisotropy and microstructural variability observed in biological tissues, which cannot be fully described using single-direction fiber assumptions. By integrating the stress contributions from fibers distributed across all orientations, weighted by the PDF, these models yield more physiologically accurate predictions of tissue behavior under mechanical loading [73].

An example of these models can be found in Gasser et al. [24], which is developed based on the earlier Holzapfel and Gasser [74,75] model, where fiber dispersion around preferred directions is modelled with a statistical orientation parameter $\left(\kappa \right)$, resulting in the Gasser–Ogden–Holzapfel (GOH) model. The GOH model employs a structural tensor approximation derived from a distribution that resembles the two-dimensional Von Mises distribution. This approach assumes that fiber orientations are symmetrically dispersed around a mean direction and simplifies their effect into a structural tensor of ($A=\kappa I+(1-3\kappa )a_{0}⊗a_{0}$) [23] and ($\kappa =\frac{1}{4}\int_{0}^{\pi }\rho (\Theta ){sin}^{3}\Theta d\Theta$), as listed in Supplementary Materials Table S1. The dispersion value of κ in the GOH model is defined as a scalar describing fiber dispersion. When κ closer to 0, the material is anisotropic κ=0 (aligned), $\kappa =\frac{1}{3}$ (isotropic), and $\kappa \epsilon (0,\frac{1}{3})$ (dispersed fibers) [24]. Therefore, the dispersion factor of κ allows replicating a smooth, continuous distribution of collagen fibers in the skin model.

The GOH model, originally developed to simulate the mechanical response of arterial layers, was later employed for skin modeling [75]. The total strain energy density in the GOH model is defined as the sum of the contributions from the isotropic ground matrix and the dispersed collagen fiber network. In 2012, Ní Annaidh et al. [23] characterized the mechanical properties of human cadaveric lower back skin using histological analysis to quantify the preferred collagen fiber orientations (Θ) and fiber dispersion (κ). The preferred orientation was determined through a Maximum Likelihood Estimation (MLE) fitting process of a Von Mises distribution, while the dispersion parameter κ was calculated numerically using the integral of $\kappa =\frac{1}{4}\int_{0}^{\pi }\rho (\Theta ){sin}^{3}\Theta d\Theta$. Therefore, the mean preferred fiber orientations of Θ=88±8 (Langer’s line orientation of 90°) and Θ=0±5 (Langer’s line orientation of 0°), were extracted from an 81-year-old female and an 89-year-old male, with corresponding dispersion values of κ=0.1535 and κ=0.1456, respectively. However, the angle γ which corresponds to the preferred orientation of the two fiber families, was estimated by half the distance between two peaks of the histogram of collagen fiber orientations, equal to 41°, consistent with histological analysis [23].

The remaining parameters of the GOH model *k*_1_, *k*_2_, *μ* and *C* was optimized through least squares curve-fitting in MATLAB, based on the stress-stretch response of experimental data. These parameters correspond to the fiber stiffness coefficient, fiber nonlinearity (stiffening) parameter, shear modulus, and bulk modulus. Although a coefficient of correlation of *R*^2^ = 99.5% and 98% for transverse and longitudinal directions were reported. Yet, an important critique can be made regarding the results presented in this study. The identical value *k*_1_, *k*_2_, *μ* and *C* (as presented in Table 2 of [23]) were reported for two different orientations of an identical skin sample (see Figure 10 in [23]). In contrast, the κ values were estimated from two individuals, an 81-year-old female and an 89-year-old male. Given the anisotropic nature of skin, it is unrealistic to expect identical material parameters for two different skin samples. This raises the possibility that the uniaxial tensile test was performed on a single skin sample from one of these individuals, with an identical κ parameter.

**Figure 7:**
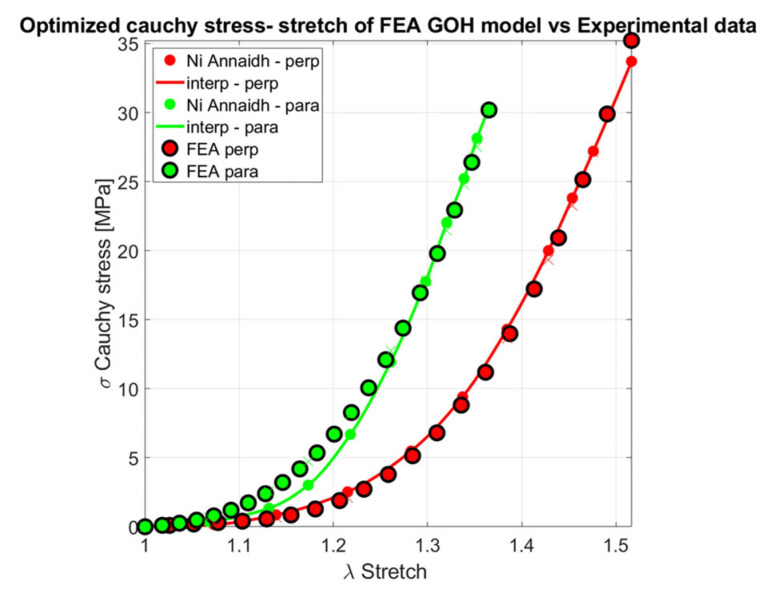
Optimized Cauchy stress–stretch curves under in vitro uniaxial test on lower back human cadaver skin in the longitudinal (green ‖) and transverse (red ⊥) directions. The FEA-fitted model is shown as circular markers, and the Experimental data are shown by solid lines (experimental data are replotted from Figure 10 in [23]).

To address this point, an independent inverse optimization analysis was conducted using MATLAB R2023a, employing the Levenberg–Marquardt algorithm (DEMO_febio_0089_iFEA_goh_skin_01 [76]). This method is well-suited for solving non-linear least squares problems, such as those encountered in the constitutive modeling of biological tissues. By combining the advantages of gradient descent and Gauss–Newton approaches, the Levenberg–Marquardt algorithm enables efficient convergence even when initial guesses are far from the optimal solution [77]. This makes it especially suitable for fine-tuning complex models like the unconstrained GOH formulation. In this optimization, which was linked to FEBio, the parameters *k*_1_, *k*_2_, *μ* and *K* were estimated using a fixed γ=41°, where bulk modulus *K* was defined as *K* = 1000*c*, ensuring the incompressibility of the skin model. The optimized parameters differed significantly from those reported by Ní Annaidh et al. [23], as shown in Table 2. Nevertheless, the optimized model with five parameters demonstrated a good fit to experimental data obtained from the lower back region, as shown in Figure 7, while accurately capturing the large stretches, which is particularly important for applications such as skin meshing.

Jor et al. [78] proposed a hyperelastic model based on a structural viscoelastic fiber-matrix model of Lanir’s work [79], excluding the viscoelastic term. Jor’s model incorporated six terms of collagen stiffness $\left(K_{c}\right)$, ground matrix stiffness $\left(K_{m}\right),$ mean fiber orientation $\left(\mu_{\theta }\right)$, fiber distribution $\left(\sigma_{\theta }\right)$, mean fiber undulation ($\mu_{x})$, and the standard deviation (SD) of the undulation distribution ${(\sigma }_{x})$, listed in Supplementary Materials Table S1. This model was developed based on Von Mises’ distribution $\left(R\left(\theta \right)\right)$ and the undulation of the fiber is governed by the Gaussian distribution of D(x) was employed to replicate the in vivo equibiaxial tension at the torso midline in pig skin. The limitation of this model is its greater sensitivity to collagen fiber orientation, which ranged from 2° to 13°, inconsistent with the mean collagen fiber orientation of 41° in histological analysis.

In addition, Meijer et al. [80] developed a structural model inspired by Lanir’s framework [79] to simulate an in vivo uniaxial test on human forearm skin. This model excluded viscoelastic terms and focused on fitting the collagen fiber stiffness, the mean undulation of the collagen fibers, and two parameters that fit the distribution of the collagen fibers. The remaining parameters were set using values from existing literature. Their fitted results fell within the range of previous studies. They conclude that the applied strains in the experiments were not high enough to engage a sufficient portion of the collagen fibers. As a result, the estimated collagen stiffness (between 51 MPa and 86 MPa) was less reliable than the other parameters [63].

Due to the complexity of accurately modeling the anisotropic mechanical properties of skin, a simplified orthotropic model has also been adopted in the literature. This approach assumes that the mechanical properties of skin vary independently along three perpendicular directions [63]. An example is provided by Bischoff et al. [81], who extended the eight-chain model originally developed by Arruda and Boyce [82], which is a constitutive model designed to capture the nonlinear, large-strain response of rubber-like materials based on non-Gaussian statistical mechanics. The eight-chain constitutive equation represents material behavior using a network of polymer-like chains arranged within a cubic unit cell. An additional prestress term is used to account for the initial tension present in the skin’s microstructure. However, this modified model failed to reproduce the stress–stretch response observed in experimental data from healthy and hypertrophic scar (HTS) skin, suggesting that it inadequately represents the orthotropic nature of skin. While the prestress term aimed to approximate directional behavior, the model did not explicitly capture the inherent anisotropy of the skin structure.

To address the limitation of reproducing the orthotropic response of the skin model, Bischoff proposed two distinct approaches. In the first method, Bischoff et al. [83] introduced an eight-chain network using an orthotropic unit cell dimension with additional terms of repulsion and entropy to account for fiber interactions and the near incompressibility of dermal tissue. With six parameters, this model reproduced the stress-stretch mechanical response of uniaxial test data on rabbit skin from Lanir and Fung. The second approach, in which the viscoelastic is incorporated, is further explained in the viscoelastic constitutive subsection.

Flynn et al. [63] (2014) highlighted that incorporating continuous collagen fiber undulation using statistical distributions, particularly those based on Lorentzian [84] or log-logistic [85] may increase computational complexity due to their mathematical formulation. Consequently, the next constitutive model was developed based on discrete fiber-based representations, as elaborated in the following section.

#### 5.1.3. Discrete Fiber-Based Models 

As explained in the previous section, continuous fiber-based models using probability density functions (PDFs) may increase computational complexity. To address this limitation, Flynn et al. [86] proposed a hyperelastic model based on six discrete weighted fiber bundles. This approach captures collagen fiber undulation using analytically integrable triangular functions. The elastin fibers are represented by a modified neo-Hookean strain energy function that prevents fiber compression. The model incorporates up to ten parameters, which were fitted to experimental data from biaxial tensile tests on rabbit abdominal skin of Fung [38] (with an error of 8.7%) and uniaxial tensile tests on pig skin (with an error of 7.6%). These parameters are directly associated with the mechanical stiffness of the collagen and elastin fibers, and a set of weights that define the orientation of the fiber bundles within the tissue. However, a limitation of the six-fiber structural framework lies in its inability to reproduce isotropic mechanical behavior when all fiber weights are assigned equally, thereby limiting its reliability in isolating anisotropic effects through weight variation alone [63].

To overcome this limitation, Flynn and Rubin [87] introduced a discrete-fiber model grounded in a generalized strain invariant. In this formulation, when fiber weights are equal, the generalized invariant reduces to an isotropic form, enabling the model to naturally exhibit isotropic behavior, even if the strain energy function is nonlinear. As a result, any variation in fiber-bundle weights can be interpreted as a direct measure of anisotropic mechanical response. This model closely matches various soft-tissue experimental data across different loading conditions. Nevertheless, a notable drawback of the generalized invariant formulation is the coupling of responses among fiber bundle systems, which complicates the interpretation of individual orientation and undulation distributions. Furthermore, the material parameters κ and $\gamma_{m}$ (stiffness and nonlinearity coefficient) are not measurable or associated with the underlying structure of biological soft tissue, making it difficult to establish reliable initial estimates for parameter optimization procedures. The generalized strain invariant model with fifteen parameters effectively replicated the mechanical behavior of biaxially loaded rabbit skin and uniaxially loaded pig skin, achieving fitting errors of approximately 12% and 16.6%, respectively. 

Prior to this model, Holzapfel and Gasser [75] established a constitutive model known as Holzapfel-Gasse-Ogden (HGO), which is implemented in the open-source finite element software FEBio to simulate the mechanical response of arterial layers. This model incorporates fixed discrete fibers to represent the structural anisotropy of soft tissues, such as skin, embedded within an isotropic Neo-Hookean ground matrix.

In addition, Groves et al. [88] developed a constitutive model based on the work of Weiss et al. [89], which is governed by a transversely isotropic, nonlinear, and incompressible constitutive law that captures the directional mechanical response of discrete collagen fiber families. The constitutive model by Weiss et al. [89] was originally established to reproduce the mechanical response of biological tissues such as ligaments, tendons, and cardiac muscle, where collagen fibers are predominantly aligned with minimal angular dispersion. The undulated behavior of the network of collagen fibers employs an exponential stress–strain relationship specific to fiber stretch. Groves extended this model by embedding it within an isotropic hyperelastic ground matrix defined by the Veronda–Westmann model [90], which was originally developed to capture the nonlinear uniaxial response of whole cat skin, using an exponential strain energy function. Consequently, the combined model successfully reproduced the anisotropic mechanical behavior observed during uniaxial tensile testing of in vitro human skin from the inferior breast region and murine skin. Although Groves et al.’s model demonstrated good agreement with experimental data, a major limitation was the optimization of fourteen material parameters independently. Additionally, to represent anisotropy, the finite element mesh was divided into three layers, with each layer assigned to a distinct fiber family oriented in different directions (longitudinal, transverse, and 45°). While this approach effectively captured directional mechanical responses, it significantly increased model complexity and computational cost.

### 5.2. Viscoelastic Constitutive Models

This section also categorizes the viscoelastic model into two types: probability density function (PDF) and discrete constitutive-based models. 

#### 5.2.1. Viscoelastic Probability Density Function Models

As discussed in Section 5.1.2, two approaches were adopted by Bischoff to address the limitations encountered in reproducing the orthotropic response of the skin model. These limitations were attributed to the inadequacy of only employing the Arruda–Boyce model with pre-stress as an anisotropic term to replicate the experimental data. The second approach proposed by Bischoff et al. [91] extended their earlier orthotropic hyperelastic model [83] by incorporating a viscoelastic term to capture time-dependent behavior and anisotropy, listed in Supplementary Materials Table S1. The model introduced two parallel orthotropic eight-chain fiber networks: one representing stable collagen fibers (elastic), and the other modeling fibers gradually reorienting within the ground substance (viscoelastic). The viscoelastic response was modelled using reptation theory, derived from polymer physics, which assumes longitudinal motion of the fibers. The orthotropic-viscoelastic model was simplified by equalizing the dimensions of fiber unit cells. This enhanced model, comprising fifteen parameters, simulated the nonlinear, anisotropic, and viscoelastic response of rabbit skin reported by Lanir and Fung [38] achieving approximately 30% error and closely matching the stress relaxation test.

Before Bischoff mode, Lanir [72,79] proposed a nonlinear, anisotropic, and viscoelastic fiber–matrix model for biological tissue. The collagen and elastin fibers are embedded in a ground matrix. Fiber orientations are represented by the distribution function of $R_{k}\left(\theta \right)$ which is governed by a Von Mises-type distribution, where each fiber type k is directionally distributed [78]. Collagen fibers are assumed to be initially undulated and to begin bearing load and straightening only after sufficient stretching. The undulated collagen fiber orientations of θ for are unequal. The distribution of collagen undulations with respect to orientation angle θ is governed by a Gaussian function D(x); which imposes a symmetric fiber profile, contrasting with the asymmetrical behavior typically observed in biological tissues [63]. A key limitation of Lanir’s viscoelastic model is the lack of a strain energy function. Stress is computed via hereditary integrals rather than being derived from an energy function, making the model non-conservative and incompatible with energy-based numerical methods, such as variational or finite element formulations.

Shoemaker et al. [92] proposed a constitutive model structurally inspired by Lanir [79], wherein the total stress in soft tissue was modeled as the combined effect of elastin and collagen fiber components. The elastin term was described using a linear viscoelastic law with a relaxation function, and the collagen fiber components also incorporated viscoelasticity with orientation-dependent behavior. A key limitation of this model was the assumption of a constant collagen fiber orientation distribution, which oversimplified the anisotropic structure of soft tissue. The parameter of this model was not directly linked to the measurable underlying structure of skin tissue [63]. Model fitting was carried out for a biaxial experiment on the in-plane elbow region of a 54-year-old female subject, and resulted in good agreement in individual tests, but poor parameter consistency across different loading conditions.

Vassoler et al. [93] extended HGO model by introducing a variational viscoelastic framework for fiber-reinforced soft tissues, enabling an additive decomposition of the strain energy into isotropic and fiber contributions, listed in Supplementary Materials Table S1. The fiber behavior was governed by the HGO-type exponential model, while the isotropic ground matrix was described by the Hencky formulation. Viscoelastic effects were incorporated through a Maxwell-type rheological model with logarithmic strain-based evolution laws, which were solved by minimizing internal variables using a Newton–Raphson method. The model, which was fitted to experimental uniaxial extension data from mouse skin under both monotonic and cyclic loading, is limited by its simplified single-fiber representation, as it fails to capture multi-directional anisotropy and relies on fitted parameters that are not directly measurable or explicitly linked to the underlying skin microstructure.

#### 5.2.2. Discrete Viscoelastic Constitutive Models

In addition to developing viscoelastic constitutive models based on probability density functions, simpler models using discrete viscoelastic elements have also been discussed in the literature. Such models were developed by Barbenel and Evans [94] and by Rubin et al. [95]. The Barbenel and Evans model is a discrete, linear viscoelastic model designed to replicate the mechanical behavior of human skin. Evans’s model focused solely on fitting stress-relaxation and creep test data, employing a combination of spring–dashpot elements that resembles a generalized Voigt structure. This structure enables modelling of time-dependent deformation and captures creep and stress relaxation. However, this model did not account for the anisotropic or nonlinear skin characteristics, and the use of discrete viscoelastic elements poses limitations due to potential inconsistency between relaxation and dynamic data, non-unique parameter identification, and sensitivity to the analysis method.

Rubin et al. [95] also developed a constitutive model incorporating a single fiber orientation, resulting in only partial representation of the skin’s anisotropic behavior. The model includes fourteen material parameters, grouped into three categories: Elastic (seven), Dissipative (three), and Hardening and Recovery (four), as listed in Supplementary Materials Table S1. The elastic parameters capture responses to volumetric, distortional, and fiber-induced deformations; the dissipative parameters model time-dependent hysteresis; and the hardening and recovery terms account for fluid-driven microstructural changes. While the model showed good agreement with uniaxial tensile data from facial skin, it failed to capture the multi-directional anisotropy characterizing facial skin tissue.

### 5.3. Quasi-Linear Viscoelastic (QLV) Constitutive Models

The other category of constitutive model is the quasi-linear viscoelastic (QLV) model, which combines a non-linear elastic response with a linear viscoelastic relaxation function. Unlike fully non-linear viscoelastic models, which have both elastic and time-dependent components that are nonlinear, QLV simplifies the time-dependent part, enabling more tractable modeling while capturing essential features such as hysteresis and rate dependence. This approach is particularly useful for biological tissues, where the elastic behavior is highly non-linear, but the viscoelastic response can be approximated linearly over time [91,96].

Lokshin and Lanir [97], extended the earlier structural framework developed by Lanir [79] introducing a comprehensive microstructural model that captures the nonlinear, anisotropic, viscoelastic, and pre-conditioning behavior of skin. The model represents skin as a composite of collagen and elastin fibers embedded in an incompressible ground matrix that exerts only hydrostatic pressure on the isotropic stress response and does not store strain energy. At low strain levels, the mechanical response is primarily governed by elastin, while at large strains, both elastin and collagen fibers significantly impact. Fiber networks are governed by a quasi-linear viscoelastic (QLV) formulation with a normalized bi-exponential relaxation function. Pre-conditioning is described through the Mullins effect for elastin, reflecting a reduced elastic stiffness reduction with cyclic loading. The model, comprising 30 structural parameters plus pressure terms, was fitted to biaxial tensile data of rabbit abdominal skin and closely matched the experimental results. Sensitivity analysis identified collagen’s non-linear elasticity, viscoelasticity, fiber undulation, and pre-conditioning as the most influential features, while elastin’s linear modulus had the least impact. Although highly accurate, the model’s complexity and computational demand limit its practicality for skin meshing simulations.

Furthermore, Bischoff [98] simplified the eight-chain fiber unit cell framework [83,91] by incorporating viscoelasticity directly at the fiber level using quasi-linear viscoelasticity (QLV) theory, listed in Supplementary Materials Table S1. This model successfully captured anisotropy and viscoelastic behavior in soft tissues and was validated against stress relaxation data from porcine skin.

In addition, Flynn et al. [99] employed Ogden constitutive model (*N* = 2), combined with various directional pre-stresses using a quasi-linear viscoelastic model incorporating a single-term Prony series. This approach was used to model in vivo facial skin mechanics under large deformation, along with a micro-robotic device that applied three-dimensional skin deformations, offering a realistic representation of large facial skin deformations. This model was limited to an intrinsically isotropic framework and accounts for anisotropy only by adding pre-stress. Based on a normalized force-displacement, the reported fit error ranged between 12% and 23%. The probe orientations were defined by θ and ϕ. For example, θ = 90°and ϕ = 0° correspond to the in-plane transverse direction, θ = 0°, ϕ = 90° to the out-of-plane normal direction, and θ = 90°, ϕ = 0°to the out-of-plane diagonal direction.

### 5.4. Summary of Constitutive Models and Transition to Damage Mechanics

**Figure 8:**
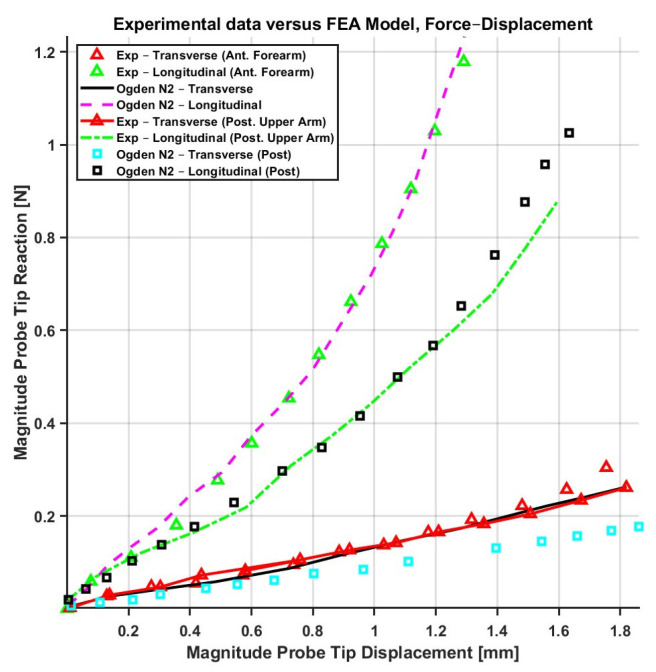
Comparison of experimental and model force–displacement responses in longitudinal and transverse directions for the average anterior forearm and posterior upper arm under in vivo loading using a force-sensitive micro-robot. For the anterior forearm, the same experimental data are compared with fitted Ogden (*N* = 2), and Tong and Fung models. Experimental data are presented alongside corresponding Ogden and Fung model results for the posterior upper arm.

Considering the elaborated existing constitutive models, several studies have attempted to compare and evaluate their performance. For instance, Flynn et al. [70] assessed the fitting accuracy of the Ogden model (*N* = 2) with extra QLV terms proposed by Fung [96] and classical Fung models against in vivo experimental data obtained from human skin on the anterior forearm and posterior upper arm using a force-sensitive micro-robot. The Fung model exhibited slightly better performance than the isotropic Ogden model, particularly in capturing the directional mechanical behavior of skin. The average error for the probe tip reaction-displacement fit was reported as 19.2% for the Ogden model, while for the same case, it was 19% for the Fung model in the anterior upper arm (shown in Figure 8).

**Figure 9:**
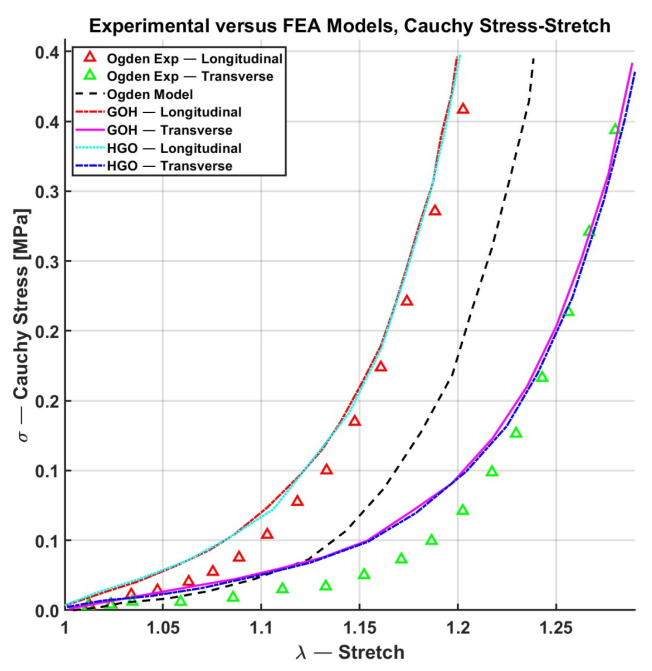
Comparison of fitted Cauchy stress–stretch curves under in vitro equibiaxial test on the lower back of human skin with Ogden, HGO, and GOH models in the longitudinal and transverse directions. Experimental data are shown as circular markers, and the FEA fitted model by solid lines (data are replotted from Figure 2 [100]).

Aldieri et al. [100] evaluated the performance of the classical Ogden model compared to the HGO and GOH models for fitting equibiaxial tensile data obtained from a human’s lower back. This test stretches the skin sample in head-to-tail (Cranio-Caudal, CC) and side-to-side (Medio-Lateral, ML) directions. The GOH model incorporates a negligible fiber dispersion parameter (*κ* = 0.0005) to avoid computational challenges, resulting in fiber alignment behavior like that of the HGO model. Consequently, both models exhibited nearly identical Normalized Mean Square Error (NMSE) values of 0.99 for the GOH model and 0.98 for the HGO model. HGO and GOH outperformed the Ogden model, as shown in Figure 9.

**Figure 10:**
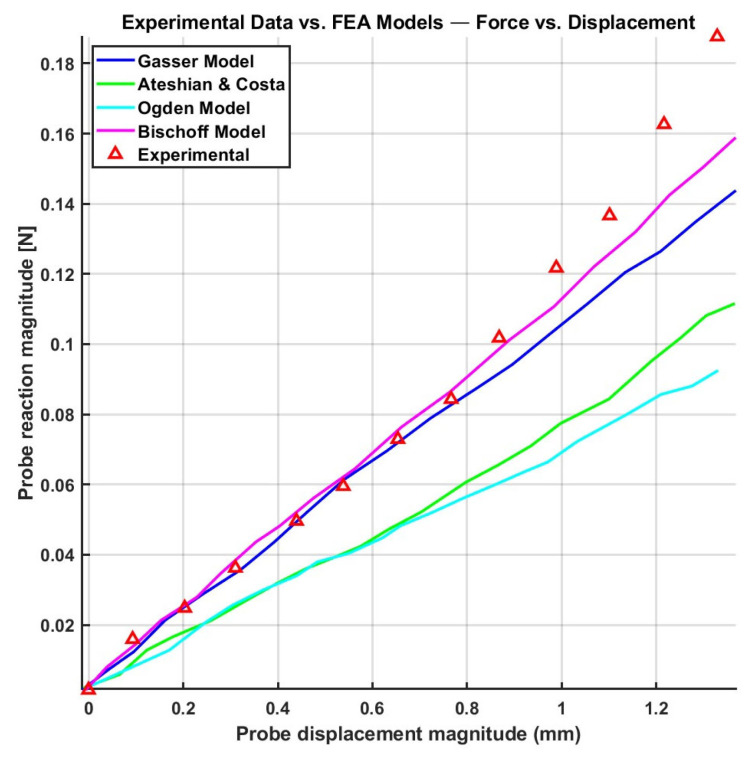
Comparison of fitted Force–Displacement under in vivo microrobot test of forehead skin fitted with Ogden, Fung, GOH, and Bischoff models (data are replotted from Figure 8 in Flynn et al., 2017 [101]).

Furthermore, Flynn et al. [101] compared several constitutive models to simulate the mechanical response of human facial skin undergoing a large deformation. They considered QLV Bischoff within the assembly of eight fibers arranged within a unit cell, Fung [67], GOH [24], and Ogden [102] constitutive models. The fitted performance of Bischoff was like that of the Ogden and Fung models. In contrast, the GOH model demonstrated a poor replication of deformation compared to the other models, and its force–displacement response appeared significantly more linear. However, in the forehead region, Bischoff and the GOH model outperformed the Ogden and Fung models, as shown in Figure 10. The limitation of this paper is that all simulations were conducted using FEBio in 2018. At that time, the dispersion-based GOH formulation was not implemented in the software, and the strain-energy function employed by Flynn et al. [101] corresponds to the aligned-fiber Holzapfel–Gasser–Ogden (HGO) variant, which excludes the dispersion parameter κ. Consequently, their comparison does not effectively represent the true GOH model. 

**Figure 11:**
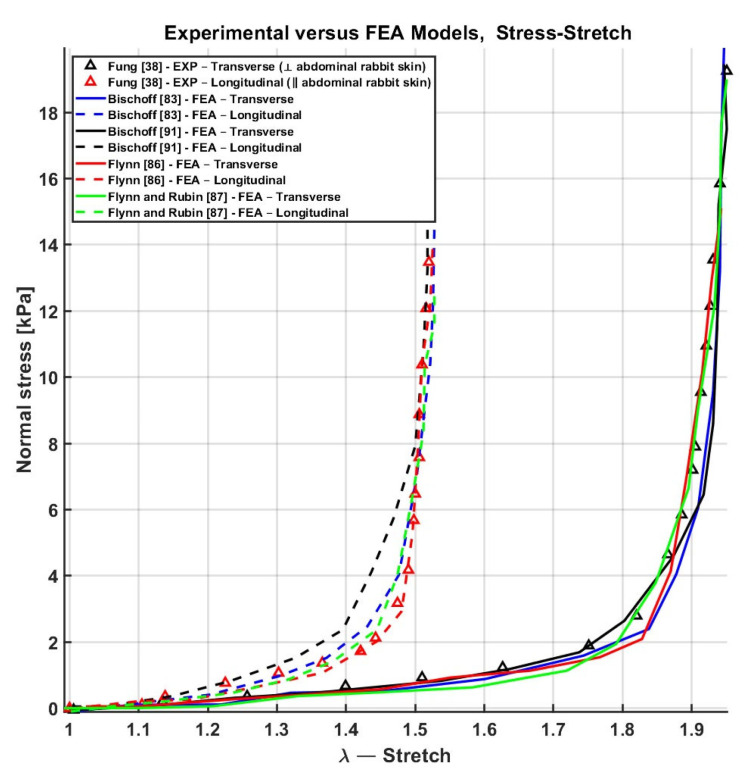
Comparison of fitted nominal stress–stretch in both longitudinal and transverse directions with fitted models of Bischoff, Flynn, and Flynn and Rubin against experimental biaxial tensile data for abdominal skin reported by Fung [38] (Data replotted from Figure 5.3 in Flynn’s work 2014 [63]).

Finally, Flynn et al. [63] compared the performance of the Bischoff [83,91], Flynn [86], and Flynn and Rubin [87] models using biaxial tensile test data originally obtained by Fung [38]. Among these, the Flynn [86] model demonstrated the best performance, with an error per fit of 10.2%. The Flynn and Rubin [87] model followed, with an error of 12.5%, while the Bischoff [91] model showed a higher error of 15.5%. In the worst case, the Bischoff model [83] yielded an error per fit of 30%. These results demonstrate that using a higher number of parameters, as in the Bischoff models [91] with 14 fitted parameters, increases model complexity and can lead to reduced fitting accuracy, as shown in Figure 11.

**Figure 12:**
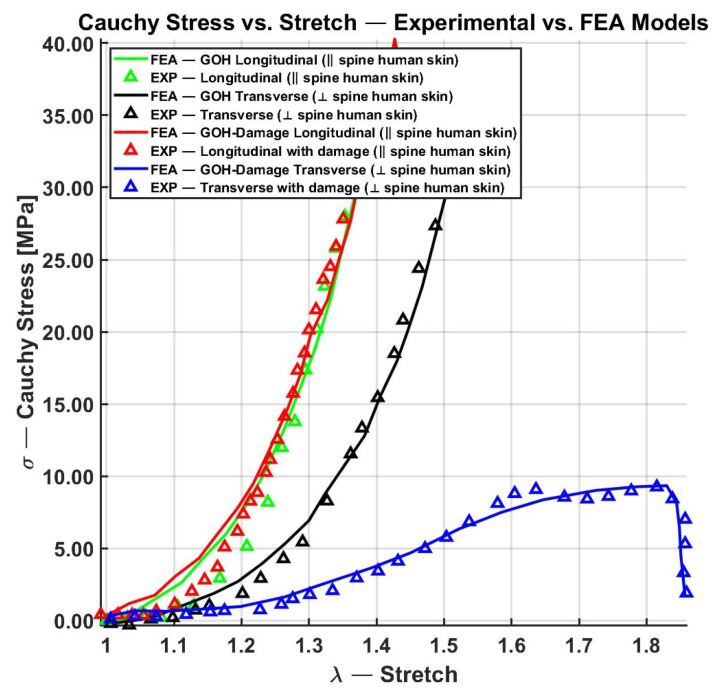
Comparison of fitted Cauchy stress–stretch curves in both longitudinal and transverse directions GOH model and damage GOH model (data are replotted from Figures 5 and 6 of [25]).

Therefore, a key aspect in selecting the best-fitted models lies in the number of parameters that are correlated with the underlying structure of skin and contribute to minimizing fitting error. Consequently, the anisotropic nonlinear GOH model, which incorporates five parameters and achieves a fitting error of less than 1%, is well-suited for our objective of simulating skin meshing under large deformations [23]. The limitation of skin damage of GOH model is further incorporated in Li and Luo [25] framework. They extended the GOH model by integrating damage mechanics inspired by Volokh’s work [103,104], enabling the simulation of fiber and matrix softening in arterial walls. This nine-parameter model with four additional damage-induced material softening of m,n,ξ, and ζ to the GOH model achieved excellent agreement with uniaxial test data of human spine skin, with R^2^ values up to 99% and fitting errors as low as 2.15% (see Figure 12). Unlike previously discussed models, this is the only one explicitly designed to capture damage progression, making it the most appropriate for skin meshing applications.

## 6. Skin Meshing Simulation and Pattern

In this section, various skin meshing patterns reported in the literature are examined, wherein only simplified isotropic constitutive models have been employed to replicate large deformations through finite element method (FEM) modeling. Accordingly, two categories of traditional skin meshing models, in conjunction with an auxetic skin meshing pattern, by which deformation is extended beyond the limitations observed in conventional configurations, are elaborated. The aim is to demonstrate that, when auxetic patterns are solely employed without consideration of the anisotropic properties of skin, a realistic mechanical response of skin models cannot be accurately reproduced.

**Figure 13:**
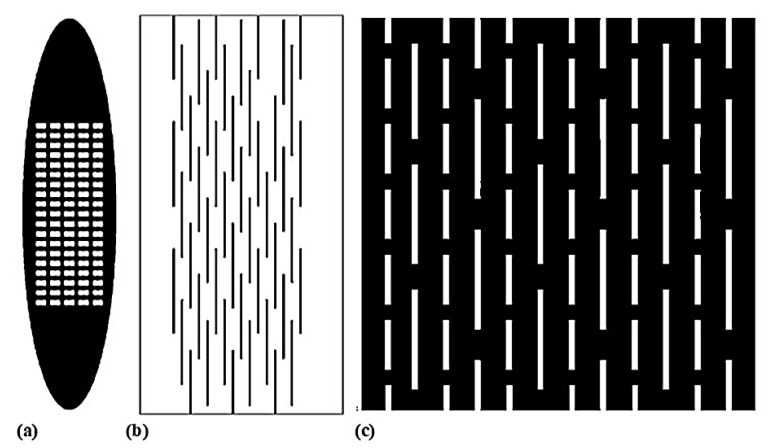
Traditional skin meshing pattern (**a**) oval skin meshing design, (**b**) Zimmer skin meshing pattern, and (**c**) 1st order skin meshing pattern.

A traditional Zimmer^®^ skin graft pattern (Czech Republic), as shown in Figure 13b, was initially employed by Capek et al. [20] to simulate the uniaxial expansion of abdominal skin from a 44-year-old female. The study was conducted to examine the influence of Langer’s lines in achieving high expansion ratios. Experimental data were obtained through uniaxial tensile testing using a Testometric M350-SCT machine on unmeshed skin samples oriented both parallel and perpendicular to Langer’s lines. Parameters for the nonlinear isotropic Yeoh model were individually derived from both datasets and subsequently applied in the simulations. The computed expansion ratios failed to reach the expected meshing ratio of 1.5:1, achieving only 1.47:1 when skin was stretched perpendicular to Langer’s line direction and 1.38:1 when skin was stretched parallel to Langer’s line direction. Nevertheless, the simulation outcomes aligned with experimental observations, confirming that greater expansion and reduced stress occurred when the skin was stretched perpendicular to Langer’s lines. These results emphasized the significance of Langer’s lines in enhancing meshing ratios but also revealed the limitations of using an isotropic model, as the anisotropic mechanical behavior of skin was not adequately captured. 

Further to this study, Gupta et al. [21], employed Yeoh model consistent with the earlier study using an alternative oval skin meshing design and horizontal slits, where the dimension and interval of slits vary, as shown in Figure 13a. This paper incorporates both directions of stretch in parallel and perpendicular to Langer’s line, employing an isotropic, fabricated, silicon-based design, which is unrealistic for representing true skin behavior. Under comparable displacement conditions, higher maximum stress was reported when the sample was stretched parallel to Langer’s lines, as opposed to the perpendicular direction. This observation aligned with the findings of Capek et al. [20], where increased stiffness was also noted in the parallel orientation.

Gupta et al. [22], also suggested the first-order traditional skin meshing model comprising two layers representing the epidermis and dermis, with material parameters for the Yeoh model adapted from the literature. The model was evaluated under both uniaxial and biaxial boundary conditions, yielding meshing ratios of 1.71 and 5.4, respectively. However, due to the absence of experimental validation and the exclusive use of the isotropic nonlinear Yeoh constitutive model, the reliability of these results remains uncertain and warrants further investigation. 

### Auxetic Design for Skin Meshing

The second category of skin meshing design, known as auxetic structure, refers to geometric and materials that exhibit a negative Poisson’s ratio, expanding laterally when stretched longitudinally [26]. This approach enables higher meshing ratios than traditional designs, provided a constitutive model accurately represents the skin’s mechanical behavior.

**Figure 14:**
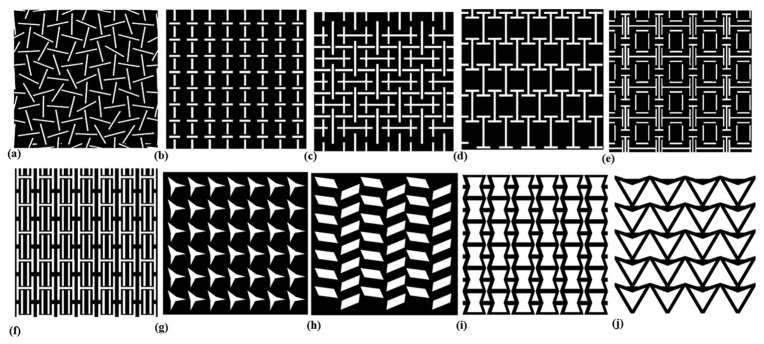
Auxetic skin pattern (**a**) Randomly oriented slits, (**b**) alternating slit (AS), (**c**) 2nd order auxetic skin meshing, (**d**) I-shaped (IS) slits, (**e**) I shape re-entrant (IRE) slits, (**f**) H-shaped (HS) slits, (**g**) rotating triangles (RT), (**h**) rotating rectangle (RR), (**i**) re-entrant honeycomb (REH), and (**j**) double arrowed honeycomb (DAH).

Initially, Grima et al. [105] found out that randomly oriented slits (shown in Figure 14a) can induce auxetic behavior, making them applicable for achieving higher meshing ratios in skin meshing. However, experimental validation was conducted solely on rubber sheets.

Gupta et al. [12,14,22,106,107,108] in a series of studies, several auxetic slit patterns were examined to monitor differences in achieved meshing ratios in skin meshing. The auxetic designs included an alternative slit pattern (AS) [12,14,22,106,107,108] (Figure 14b), Second-order AS auxetic design (Figure 14c) [12,14], I-shaped (IS) slits [14,108,109] (Figure 14d), I shape re-entrant (IRE) slits (Figure 14e) [14], H-shaped (HS) (Figure 14f) [14,106], rotating triangles (RT) (Figure 14g) [14,107], rotating rectangle (RR) (Figure 14h) [14,106], re-entrant honeycomb (REH) (Figure 14i) [14,106], and double arrowed honeycomb (DAH) (Figure 14j) [14].

The first comparison was conducted between the AS and second-order AS auxetic models, employing the Mooney–Rivlin constitutive model under uniaxial strains of up to 300%. Both models achieved R^2^ values of 0.99, in good agreement with experimental data obtained from uniaxial tension tests on fabricated silicon patterns. However, as silicon exhibits rubber-like properties, it does not accurately represent the layered structure or the complex mechanical behavior of human skin [12]. 

In the next study, Gupta et al. [14] employed two layers representing the epidermis and dermis, with material parameters for the Yeoh model adapted from the literature to calculate the stress-strain response of various auxetic patterns in skin samples under uniaxial and biaxial tension. The various designs contained alternating slit (AS), I-shaped (IS) slits, rotating triangles (RT), I shape re-entrant (IRE), H-shaped (HS) slits, rotating rectangle (RR), re-entrant honeycomb (REH), and double arrowed honeycomb (DAH), as shown in Figure 14b,d–j. At 200% strain, the highest stress under both uniaxial and biaxial loading was experienced by the RR model, while the lowest was observed in the HS and IRE models, respectively. This contrasts with the maximum negative Poisson’s ratio of –1.158 observed in the AS pattern under uniaxial tension, which should, in principle, enable superior meshing ratios compared to the RR model.

Subsequently, a two-layer, nonlinear, isotropic Yeoh constitutive model was employed, with parameters adapted from the literature to represent the epidermis and dermis layers of a skin model. Consistent with the earlier study [108], the AS and second-order AS auxetic models were utilized to assess differences under uniaxial and biaxial tension. Meshing ratios of 1.77 (traditional) and 5.36 (auxetic) under uniaxial tension, and 3.15 and 5.24 under biaxial tension, reflect the enhanced expansion performance of the auxetic models. Despite the absence of empirical validation for their skin meshing model, they proposed a composite material, comprising agarose, intralipid, a buffer solution, and deionized water, but lacking mineral content, for fabrication via bioprinting, following a concept suggested by Zhang’s work [22,110].

Additionally, they investigated AS and IS models using a two-layer nonlinear isotropic Yeoh constitutive model, emphasizing that variations in slit dimensions directly influence Poisson’s ratio, leading to greater expansion under similar uniaxial and biaxial loading conditions, with strains of up to 100% [108,109].

Finally, a rotating triangle geometry with varying internal angles was evaluated under uniaxial tension up to 50% strain [107]. Three constitutive models of Neo-Hookean, Mooney–Rivlin, and Yeoh were employed, with the Yeoh model showing the best agreement with experimental data, followed by the Neo-Hookean and Mooney–Rivlin models. An internal angle of 0° yielded the highest expansion capacity, followed by 30° (Figure 14g), while 135° resulted in the least expansion. Although the study detailed the 3D printing methodology using two elastomer materials, only silicone was used in the fabrication process. Consequently, both the material and the constitutive models used fail to replicate the realistic mechanical behavior of human skin.

## 7. Conclusions

Skin meshing was found to be the most practical approach to severe burn treatments due to its cost-effectiveness, time savings, and reduced risk of infection [11,51]. However, across clinical and computational studies, a key gap is the persistent mismatch between manufacturer-reported and clinically achieved meshing ratios [13,14,15]. This discrepancy arises from the use of simplified constitutive models in skin meshing simulations, which fail to replicate the skin’s anisotropic, nonlinear mechanical behavior during large deformations [12,20,21,22]. Thus, a single-layer model, such as the unconstrained GOH formulation, is adequate, as it embeds collagen fiber orientation and dispersion and captures Langer’s line-driven stiffness and load-bearing behavior of the dermis undergoing large deformations [111]. However, the GOH model alone does not account for skin damage. Therefore, integrating damage into the GOH model proposed by Li and Lou [25] is a suitable approach for replicating a realistic skin meshing model at large strains and slow stretching ratios.

The second reason for the discrepancy was identified as limited evaluation of slit geometry, spacing, and loading direction, all of which influence stress distribution during expansion. Auxetic meshing patterns, which enable negative Poisson ratios, have shown potential for generating higher skin expansion [12,14,22,106,107,108]. 

Therefore, future research should integrate nonlinear anisotropic models with systematically designed auxetic geometries and directly compare optimal expansions across different orientations of Langer’s lines. Additional factors such as age-related collagen changes, regional variations in dermal thickness, and differences in fiber dispersion may further refine these predictions. Such an approach could support the development of next-generation skin meshers capable of achieving higher, more reliable expansion ratios and improved clinical outcomes.

## Figures and Tables

**Table 2:** Optimized material parameters for the GOH model using the Levenberg–Marquardt algorithm.

Constitutive Model	c [MPa]	$k_{1}$	$k_{2}$	γ	κ	K
GOH (parallel, Perpendicular)	1.30259	124.351	0.00013	$41^{{}^{\circ}}$	0.2985	1302.59

## Data Availability

Data could be provided by contacting to the main author.

## Supplementary Materials

The following supporting information can be downloaded at: https://www.mdpi.com/article/10.3390/biomimetics11010004/s1, Table S1. Constitutive models for the dermis layer of skin.
