# Supplementary material for: A Comprehensive Review of Computational and Experimental Studies on Skin Mechanics and Meshing: Discrepancies, Challenges, and Optimization Strategies
Source: Biomimetics (Basel). 2025 Dec 22;11(1):4. doi: 10.3390/biomimetics11010004 (PMC12839001; doi:10.3390/biomimetics11010004)
Supplement: Supplementary file 1 [file biomimetics-11-00004-s001.zip › biomimetics-3997929-supplementary.pdf]

Table S1

Constitutive models for the dermis layer of skin

Constitutive model	Properties	Site	Strain energy function (Theoretical equation) and parameters	FEBio unconstrained Strain energy function and parameters (compressible)	FEBio constrained Strain energy function and parameters (incompressible)	FEBio coupled Strain energy function and parameters	FEBio uncoupled Strain energy function and parameters
Mooney–Rivlin	isotropic, nonlinear, hyperelastic	pig rump skin [1]	$\phi = C_1(\lambda_1^2 + \lambda_2^2 + \lambda_3^2 - 3) + C_2\left(\frac{1}{\lambda_1^2} + \frac{1}{\lambda_2^2} + \frac{1}{\lambda_3^2} - 3\right)$ [1]<br>C_1, C_2: positive constant, $C_1 \neq C_2$			$\boldsymbol{W} = c_1(I_1 - 3) + c_2(I_2 - 3) - 2(c_1 + 2c_2)\ln J + \frac{\lambda}{2}(\ln J)^2$ (nearly-incompressible) [3]<br>I_1, I_2 : the first and second invariants of the right Cauchy-Green deformation tensor $\mathbf{C}$<br>J : the determinant of the deformation gradient tensor<br>c_1, c_2: constant parameters [P]<br>κ: bulk modulus [P]	$\boldsymbol{W} = c_1(\tilde{I}_1 - 3) + c_2(\tilde{I}_2 - 3) + \frac{1}{2}K(\ln J)^2$ [3]<br>$\tilde{I}_1, \tilde{I}_2$: the first and second invariants of the right Cauchy-Green deformation tensor $\mathbf{C}$<br>J: the Jacobian of the deformation gradient tensor<br>c_1, c_2: constant parameters [P]<br>κ: bulk modulus [P]
Mooney–Rivlin	isotropic, nonlinear, hyperelastic	intestine of freshly euthanized adult Yorkshire pigs [2]	$W(I_C, II_C) = d_1(I_C - 3) + d_2(II_C - 3)$ [2]<br>I_C, II_C: the first and the second invariants of the right Cauchy–Green deformation tensor $\mathbf{C}$<br>d_1, d_2: constant parameters				
Mooney–Rivlin	isotropic, nonlinear, hyperelastic	intestine of freshly euthanized adult Yorkshire pigs [2]	$W(I_C) = c_1(I_C - 3)$ [2]<br>I_C: the first invariant of the right Cauchy–Green deformation tensor $\mathbf{C}$<br>c_1: constant parameter	$W = \frac{\mu}{2}(I_1 - 3) - \mu \ln J + \frac{\lambda}{2}(\ln J)^2$ [3]<br>I_1, I_2 : the first and second invariants of the right Cauchy-Green deformation tensor $\mathbf{C}$<br>J : the determinant of the deformation gradient tensor<br>$\mu = \frac{E}{2(1+\nu)}$<br>$\lambda = \frac{\nu E}{(1+\nu)(1-2\nu)}$<br>$E$: Young's modulus [**P**]<br>ν: Poisson's ratio			
Ogden (N=1)	isotropic, nonlinear, hyperelastic	pig rump skin [1]	$\phi = \frac{2\mu}{\alpha^2}(\lambda_1^\alpha + \lambda_2^\alpha + \lambda_3^\alpha - 3)$ [1] (N=1)<br>$\lambda_i (i = 1,2,3)$: principal stretch ratios<br>α: strain hardening exponent<br>μ: shear modulus under infinitesimal straining		$W = \sum_{i=1}^{N} \frac{c_i}{m_i^2}\left(\tilde{\lambda}_1^{m_i} + \tilde{\lambda}_2^{m_i} + \tilde{\lambda}_3^{m_i} - 3\right) + U(J)$ [3]<br>$\tilde{\lambda}_i^2$: the eigenvalues of $\tilde{\mathbf{C}}$<br>c_i, m_i: material coefficients<br>κ: bulk modulus [P]	$W(\lambda_1, \lambda_2, \lambda_3) = \frac{1}{2}c_p(J-1)^2 + \sum_{i=1}^{N} \frac{c_i}{m_i^2}\left(\lambda_1^{m_i} + \lambda_2^{m_i} + \lambda_3^{m_i} - 3 - m_i \ln J\right)$ [3]<br>λ_i^2: the eigenvalues of the right or left Cauchy deformation tensor<br>c_p,c_i<br>m_i: material coefficients<br>c_p:Bulk-like modulus [**P**]	
Ogden (N=1,2)		anterior and posterior forearm [4]	$W = \sum_{i=1}^{N} \frac{2\mu_i}{\alpha_i^2}\left(\lambda_1^{\alpha_i} + \lambda_2^{\alpha_i} + \lambda_3^{\alpha_i} - 3\right) + p(J-1)$ [4]<br>N, μ_i, and α_i: constant parameters<br>$\lambda_i (i = 1,2,3)$: the principal stretches<br>p: Lagrange multiplier representing partial of hydrostatic pressure<br>J: the volume ratio				
Tong and Fung	nonlinear, anisotropic, hyperelastic	rabbit abdominal skin [5]	$W = \frac{1}{2}(\alpha_1 e_1^2 + \alpha_2 e_2^2 + 2\alpha_4 e_1 e_2) + \frac{1}{2}c \exp(a_1 e_1^2 + a_2 e_2^2 + a_3 e_{12}^2 + 2a_4 e_1 e_2 + \gamma_1 e_1^3 + \gamma_2 e_2^3 + \gamma_4 e_1^2 e_2 + \gamma_5 e_1 e_2^2)$ (2D orthotropic [5])<br>7 parameters (when $\gamma_i = 0$), 10 parameters	$\Psi = \frac{1}{2}c(e^Q - 1) + U(J)$, (3D orthotropic [7], [8])<br>$Q = c^{-1}\sum_{a=1}^{3}[2\mu_a \mathbf{M}_a : \mathbf{E}^2 + \sum_{b=1}^{3}\lambda_{ab}(\mathbf{M}_a : \mathbf{E})(\mathbf{M}_b : \mathbf{E})]$,	$\tilde{\Psi} = \frac{1}{2}c(e^Q - 1) + U(J)$ (3D orthotropic [7], [8])<br>$\tilde{Q} = c^{-1}\sum_{a=1}^{3}\left[2\mu_a \mathbf{M}_a : \tilde{\mathrm{E}}^2 + \sum_{b=1}^{3}\lambda_{ab}\left(\mathbf{M}_a : \tilde{\mathbf{E}}\right)\left(\mathbf{M}_b : \tilde{\mathbf{E}}\right)\right]$,		

			(with γ_i)<br>α_1, α_2: Exponential polynomial coefficients<br>α_4: Coupling term coefficient<br>$a_i (i = 1,2,3)$: Quadratic terms<br>a_4: coupling between e_1 and e_2<br>$a_i (i = 1,2,3,4)$: Coefficients in the polynomial part<br>c: scales the exponential nonlinearity<br>γ_i: higher-order anisotropic and cross-product terms	$U(J) = \frac{\kappa}{2}(\ln J)^2$, $\mathbf{E} = (\mathbf{C} - \mathbf{I})/2$, $\mathbf{M} = \mathbf{V}_a \otimes \mathbf{V}_a$<br>$\mathbf{V}_a$: the initial direction of material axis a<br>$\mu_a (a = 1,2,3)$: Lamé constants<br>$\lambda_{ab} = \lambda_{ba}$ $(a = 1,2,3)$ are related to Young's modulus of E_a, shear modulus of G_{ab}, and Poisson's ratios ν_{ab}<br>$E_a (a = 1,2,3)$: Young's modulus [P]<br>$G_{ab} (ab = 12,23,31)$: shear modulus [P]<br>$\nu_{ab} (ab = 12,23,31)$: Poisson's ratio<br>c: coefficient [P]<br>κ: bulk modulus [P]	$\tilde{\mathbf{E}} = \frac{1}{2}(\tilde{\mathbf{C}} - \mathbf{I})/2$, $\mathbf{M} = \mathbf{V}_a \otimes \mathbf{V}_a$<br>$\mathbf{V}_a$: the initial direction of material axis a<br>$\mu_a (a = 1,2,3)$: Lamé constants<br>$\lambda_{ab} = \lambda_{ba}$ $(a = 1,2,3)$ are related to Young's modulus of E_a, shear modulus of G_{ab}, and Poisson's ratios ν_{ab}<br>$E_a (a = 1,2,3)$: Young's modulus [P]<br>$G_{ab} (ab = 12,23,31)$: shear modulus [P]<br>$\nu_{ab} (ab = 12,23,31)$: Poisson's ratio<br>c: coefficient [P]<br>κ: bulk modulus [P]		
Tong and Fung	nonlinear, anisotropic, hyperelastic	intestine of freshly euthanized adult Yorkshire pigs [2]	$W = \frac{1}{2}c(e^Q - 1)$ [2]<br>$Q(E) = a_1 E_{11}^2 + a_2 E_{22}^2 + 2a_3 E_{11} E_{22}$<br>$c, a_1, a_2, a_3$: material parameters				
Tong and Fung	nonlinear, anisotropic, hyperelastic	human facial skin [6]	$W = \frac{1}{2}c(e^Q - 1) + U(J)$ [6]<br>$Q = c^{-1} \sum_{a=1}^{3} [2\mu_a a_a^0 \otimes a_a^0 : \bar{E}^2 + \sum_{b=1}^{3} \lambda_{ab} (a_a^0 \otimes a_a^0 : \bar{E})(a_b^0 \otimes a_b^0 : \bar{E})]$<br>$c$: stiffness parameter<br>$\mu_a, \lambda_{ab}$: Lam´e parameters<br>$\bar{E} = \frac{1}{2}(\bar{F}^T \bar{F} - I)$: the deviatoric Green-Lagrange strain tensor<br>a_a^0: defines an initial direction of a material axis a				
Holzapfel Gasser Ogden (HGO) [9]	anisotropic, nonlinear, hyperelastic	arterial wall [9]	In this simulation, the isotropic matrix was modelled using a Neo-Hookean formulation, while an exponential function was employed to represent the anisotropic response. The mechanical contributions of the two embedded fiber families were considered decoupled within the anisotropic component of the model [9].<br>$\Psi(I_1, I_4, I_6) = \Psi_{isotropic}(I_1) + \Psi_{anisotropic}(I_4, I_6)$<br>$\Psi_{isotropic}(I_1) = \mu(I_1 - 3)$,			$\boldsymbol{W}_{isotropic} = c_1(I_1 - 3) + c_2(I_2 - 3) - 2(c_1 + 2c_2)\ln J + \frac{\lambda}{2}(\ln J)^2$ (nearly-incompressible) [3]<br>I_1, I_2 : the first and second invariants of the right Cauchy-Green deformation tensor $\mathbf{C}$<br>J : the determinant of the deformation gradient tensor<br>c_1, c_2: constant parameters [P]	$\boldsymbol{W}_{isotropic} = c_1(\tilde{I}_1 - 3) + c_2(\tilde{I}_2 - 3) + \frac{1}{2}K(\ln J)^2$ [3]<br>$\tilde{I}_1, \tilde{I}_2$: the first and second invariants of the right Cauchy-Green deformation tensor $\mathbf{C}$<br>J: the Jacobian of the deformation gradient tensor<br>c_1, c_2: constant parameters [P]<br>κ: bulk modulus [P]

HGO	anisotropic,<br>nonlinear,<br>hyperelastic		$\Psi_{anisotropic}(I_4, I_6) = \frac{k_1}{2k_2} \sum_{\alpha=4,6} \{\exp[k_2(I_\alpha - 1)^2] - 1\}$<br>$\mu$, k_1: material parameters with dimension of stress<br>k_2: dimensionless material parameter<br><br>$W = \frac{\mu}{2}(I_1 - 3) + \mu \sum_{i=1}^{2} \frac{k_{i1}}{2k_{i2}} \{exp(k_{i2}[tr(H_i C) - 1]^2) - 1\}$ [6]<br>μ: shear modulus of ground matrix<br>ki_1, k_{i2}: material constants			κ: bulk modulus [P]<br>$\Psi_{anisotropic} = \frac{\zeta}{\alpha\beta}(\exp[\alpha(I_n - 1)^\beta] - 1)$<br>$I_n = \lambda_n^2 = n_r.\mathbf{C}.n_r$ (the square of the fiber stretch)<br>$n = \mathbf{F}.n_r/\lambda_n$<br>$\zeta$: a measure of the fiber modulus [P]<br>α : coefficient of exponential argument<br>β: power of exponential argument<br>$\Psi_{HGO} = \boldsymbol{W_{isotropic}} + \Psi_{anisotropic}$	$\widetilde{\Psi}_{anisotropic} = \frac{\zeta}{\alpha\beta}(\exp[\alpha(\tilde{I}_n - 1)^\beta] - 1)$<br>$\tilde{I}_n = \tilde{\lambda}_n^2 = n_r.\tilde{\mathbf{C}}.n_r$ (the square of the fiber stretch)<br>$n = \tilde{\mathbf{F}}.n_r/\tilde{\lambda}_n$<br>$\zeta$: a measure of the fiber modulus [P]<br>α : coefficient of exponential argument<br>β: power of exponential argument<br>$\widetilde{\Psi}_{HGO} = \boldsymbol{W_{isotropic}} + \widetilde{\Psi}_{anisotropic}$
Gasser Ogden Holzapfel (GOH)	anisotropic,<br>nonlinear,<br>hyperelastic	Human lower back [10]	$W = \frac{c}{2}(I_1 - 3) + \mu \sum_{i=1,2} \frac{k_{i1}}{2k_{2i}} \{e^{k_{i2}[tr(H_i C) - 1]^2} - 1\}$ [10]<br>With the structure tensor contribution of $tr(H_i C) = \kappa_i I_i + (1 - 3k_i) I_{4i}$, $I_{4i} \equiv a_i.C.a_i$<br>μ: shear modulus of the isotropic ground matrix [P]<br>k_{i1} : fiber stiffness in small-strain regime for fiber family i (dimensionless)<br>k_{2i}: exponential stiffening parameter for fiber family i (dimensionless)<br>κ_i:fiber dispersion parameter for family i<br>I_1 :first invariant of the right Cauchy-Green tensor (trace of C)<br>I_{4i}: Square of the stretch in the fiber direction a_i: $a_i.C.a_i$<br>H_i: structure tensor for fiber family i, dependent on κ_i and direction a_i	$\boldsymbol{\psi_r} = \frac{c}{2}(I_1 - 3) - c \ln J + \frac{k_1}{2k_2} \sum_\alpha (exp(k_2 \langle E_\alpha \rangle^2) - 1) + \frac{k_0}{2}\left(\frac{J^2 - 1}{2} - \ln J\right)$, [13]<br>E_α: fiber strain<br>$E_\alpha = \kappa(I_1 - 3) + (1 - 3\kappa)(I_{4\alpha} - 1)$<br>$I_1$: first invariant of the right Cauchy-Green deformation tensor $\tilde{\mathbf{C}}$<br>$I_1 = tr\,\mathbf{C}$, $I_{4\alpha} = a_{\alpha r}.\mathbf{C}.a_{\alpha r}$<br>$I_{4\alpha}$: second invariant associated with the fiber direction<br>c: shear modulus of ground matrix [P]<br>k_1: fiber modulus [P]<br>k_2: fiber exponential coefficient [P]<br>γ: fiber mean orientation angle [deg]<br>κ: fiber dispersion<br>K:bulk modulus [P]			$\widetilde{\boldsymbol{\psi}}_{\boldsymbol{r}} = \frac{c}{2}(\tilde{I}_1 - 3) + \frac{k_1}{2k_2} \sum_{\alpha=1}^{2} \left(\exp\left(k_2 \langle \tilde{E}_\alpha \rangle^2\right) - 1\right)$, [13]<br>$(J)$: default volumetric strain energy function<br>$U(J) = \frac{k}{2}\left(\frac{J^2 - 1}{2} - \ln J\right)$<br>$\tilde{E}_\alpha$: fiber strain in the deviatoric part<br>$\tilde{E}_\alpha = \kappa(\tilde{I}_1 - 3) + (1 - 3\kappa)(\tilde{I}_{4\alpha} - 1)$<br>$\tilde{I}_1$: first invariant of the right Cauchy-Green deformation tensor $\tilde{\mathbf{C}}$<br>$\tilde{I}_1 = tr\,\tilde{\mathbf{C}}$, $\tilde{I}_{4\alpha} = a_{\alpha r}.\tilde{\mathbf{C}}.a_{\alpha r}$<br>$\tilde{I}_{4\alpha}$: second invariant associated with the fiber direction<br>c: shear modulus of ground matrix [P]<br>k_1: fiber modulus [P]<br>k_2: fiber exponential coefficient [P]<br>γ : fiber mean orientation angle [deg]<br>κ: fiber dispersion<br>K:bulk modulus [P]
Gasser Ogden Holzapfel (GOH)	anisotropic,<br>nonlinear,<br>hyperelastic	Human lower back [11]	$W = \frac{c}{2}(I_1 - 3) + \frac{k_1}{2k_2}\left(e^{k_2(I_4 - 1)^2} - 1\right) + \frac{k_3}{2k_4}\left(e^{k_4(I_6 - 1)^2} - 1\right)$<br><br>$c$: isotropic neo-Hookean matrix stiffness parameter<br>k_1 :fiber family 1 stress-like parameter (contributes to stiffness in preferred fiber direction)<br>k_2 : dimensionless exponential growth				

			parameter (nonlinearity of fiber response)<br>k_3: fiber family 2 stress-like parameter<br>k_4 : dimensionless exponential growth parameter for second fiber family
Gasser Ogden Holzapfel (GOH)	anisotropic, nonlinear, hyperelastic	Facial skin [6]	
			$W_{Gasser} = \frac{\mu}{2}(\bar{I}_1 - 1) + \frac{k_1}{k_2}\left\{e^{k_2[\bar{I}_n(\theta)-1]^2} - 1\right\} + U(J)$ [6]<br>μ, k_1: control the stiffness of the skin at small strains<br>k_2: a dimensionless parameter that controls the stiffness at large strains<br>$\bar{I}_n(\theta) = N\bar{C}N$: fiber stretch squared of nth family
Gasser Ogden Holzapfel (GOH),	anisotropic, nonlinear, hyperelastic	Human skin [12]	
			$W = \frac{\mu}{2}\left[(I_1 - 3) - \frac{(I_1-3)^{m+1}}{(m+1)(\xi-3)^m}\right] + \frac{k_1}{k_2}\left\{exp(k_2A^2) - 1 - \frac{2k_2A^{n+2}}{(n+2)(\xi^2-1)^n}\right\}$ [12]<br>$A = \lambda_f^2 - 1\ ,\ \lambda_f = \sqrt{\kappa I_1 + (1-3\kappa)I_4}$<br>$\mu$: matrix shear modulus which controls stiffness of the isotropic ground matrix<br>k_1: fiber stiffness parameter which governs the strength of collagen fibers<br>k_2: fiber exponential parameter which controls nonlinear stiffening of fibers<br>β: mean fiber orientation angle – typically with respect to reference axis (°)<br>κ: fiber dispersion parameter<br>m: matrix damage sharpness – how abruptly the matrix begins to soften<br>n: fiber damage sharpness – how abruptly fibers soften or fail<br>ξ : matrix damage threshold – value of I_1 (or similar) at which matrix damage starts<br>ζ : fiber damage threshold – stretch at which fibers begin to fail (e.g., k_f)<br>m, n, ξ, ζ : damage-induced material softening [12]
Jor	anisotropic, nonlinear, hyperelastic	Torso midline pig skin [14]	$W_f = \sum_\theta R(\theta)\,.\,\omega_f(\lambda)\Delta\theta$ [14]<br>von Mises distribution of fiber orientation

			$R(\theta) = \frac{\exp\left[\kappa_\theta \cos\left(2(\theta-\mu_\theta)\right)\right]}{\pi I_0(\kappa_\theta)}$<br>strain energy function of the ground matrix<br>$W_m = K_m(I_1 - 3)$<br>function of the fiber stretch ratio<br>$\omega_f(\lambda) = \frac{K_c}{2}(\lambda-1)^2$<br>$\lambda = \sqrt{2\gamma_f' + 1} =$<br>$\sqrt{2(E_{11}cos^2\theta + E_{22}sin^2\theta + 2E_{12}cos\theta sin\theta) + 1}$<br>The distribution function for fiber undulation is assumed to be Gaussian and can thus be defined by:<br>$D(x) = \frac{1}{\sqrt{2\pi}\sigma_x}\exp\left\{-\frac{(x-\mu_x)^2}{2\sigma_x^2}\right\}$<br>$K_c$: collagen stiffness<br>$K_m$: ground matrix stiffness<br>μ_θ: mean fiber orientation<br>σ_θ: fiber distribution<br>μ_x: mean fiber undulation<br>σ_x: standard deviation (SD) of the undulation distribution
Groves et al	anisotropic, nonlinear, hyperelastic,	human skin [15]	$W = F_1(I_1, I_2) + F_2(\lambda) + \frac{K}{2}[\ln J]^2$ (uncoupled [15])<br>isotropic Matrix (Veronda–Westmann):<br>$F_1(I_1, I_2) = c_1\left(e^{C_2(I_1-3)} - 1\right) - \frac{C_1C_2}{2}(I_2 - 3)$<br>strain energy function for the fibers:<br>if $\lambda < 1, \lambda\frac{\partial F_2}{\partial\lambda} = 0$<br>if $1 < \lambda < \lambda_m, \lambda\frac{\partial F_2}{\partial\lambda} = C_3\left(e^{C_4(\lambda-1)} - 1\right)$<br>if $\lambda \geq \lambda_m, \lambda\frac{\partial F_2}{\partial\lambda} = C_5 + C_6\lambda$ [16]<br>$C_5 = C_3C_4\lambda\left(e^{C_4(\lambda-1)}\right)$<br>$C_1$: isotropic matrix stiffness<br>C_2: exponential stiffening rate for matrix<br>C_3: scales exponential stress in fiber toe region<br>C_4: rate of uncrimping of fiber<br>C_5: modulus of fully straightened fibers<br>C_6: optional further linear contribution (often omitted)<br>λ_m: stretch at which fibers are fully straightened<br>a_i: fiber orientation vector (angle or direction) for each fiber family<br>K: bulk modulus

Vassoler	nonlinear, anisotropic, viscoelastic	mouse skin [17]	$\Psi = \Psi_{isotropic} + \Psi_f$ [17]<br>$\Psi_{isotropic}(\mathbf{F}_{n+1}; \xi_n) = \Delta\varphi(\hat{\mathbf{C}}_{n+1}) + \Delta U(J_{n+1}) + \min_{M_j^{\nu}.\Delta q_j^{\nu}} \{\Delta\varphi^e(\hat{\mathbf{C}}^e_{n+1}) + \Delta t \psi(\mathrm{D}^{\nu}_{n+1})\}$<br>$\varphi, \varphi^e$: Hencky-type strain energy functions (quadratic in logarithmic strain)<br>$U(J) = \frac{k}{2}(\ln J)^2$: the volumetric term<br>$\psi$: the viscous potential in terms of eigenvalues of the viscous stretch rate<br>$\Psi_f = \Delta\varphi(\lambda_{f_{n+1}}) + \min_{\Delta\varepsilon_f^{\nu}} \left\{\varphi_f^e\left(\varepsilon_f^e(\Delta\varepsilon_f^{\nu})\right) + \Delta t \psi_f\left(\frac{\Delta\varepsilon_f^{\nu}}{\Delta t}\right)\right\}$<br>$\varphi_f^e$: Holzapfel-type exponential function<br>ψ_f: Hencky-type dissipation function<br>μ: linear stiffness of isotropic Hencky strain energy (Matrix shear modulus)<br>κ: Controls compressibility (volumetric term), (Bulk modulus)<br>k_1: Scales fiber stress (elastic exponential term), (Fiber stiffness coefficient)<br>k_2: Controls shape of stress–strain fiber curve, (Fiber nonlinearity)<br>ν: Viscous resistance in fiber Maxwell branch, (Fiber viscosity)
Rubin-Bodner	nonlinear, anisotropic, viscoelastic	Superficial Musculoaponeurotic System (SMAS) and facial human skin [18]	$w = \frac{\mu_0}{2q}[\exp(qg) - 1]$ [18]<br>$g = g_1(J) + g_2(\beta_1) + g_3(\lambda_1) + g_4(\alpha_1)$<br>$g_1(J) = 2m_1[(J-1) - \ln(J)]$ (volumetric)<br>$g_2(\beta_1) = m_2(\beta_1 - 3)$ (elastic distortion)<br>$g_3(\lambda_1) = \frac{m_3}{m_4}(\lambda_1 - 1)^{2m_4}$ (fiber stretch)<br>$g_4(\alpha_1) = \alpha_1 - 3$ (dissipative distortion)<br>J: volumetric deformation<br>β_1: invariant of elastic distortional deformation<br>λ_1: stretch in the single fiber direction<br>α_1 : invariant of dissipative distortional deformation<br>with $\langle x \rangle = \frac{1}{2}(x + \|x\|)$<br>Elastic parameters<br>$\mu_0$: nonlinear shear modulus<br>q: exponential stiffness factor (nonlinearity)<br>k_1 [GPa]: volumetric stiffness (bulk modulus)<br>m_2: distortional stiffness weight<br>m_3: fiber stiffness weight<br>m_4: fiber nonlinearity exponent

			Dissipative parameters<br>Γ_1 (s^{-1}): dissipation (rate-independent term)<br>Γ_2: dissipation (rate-dependent term)<br>n: transition sharpness (rate sensitivity)<br>Hardening recovery parameters<br>r_1: hardening rate (during stress relaxation)<br>r_2: hardening rate (during loading)<br>r_3 (s^{-1}) : strain-rate control for hardening separation<br>r_4 (s^{-1}): recovery rate of hardening<br>r_5: recovery exponent (nonlinearity in recovery)
Flynn and Rubin	anisotropic, nonlinear, hyperelastic	rabbit abdominal skin [19]	The strain energy function W per unit mass of the collection of six fiber bundles is [19]<br>$W = f(J) + \sum_{i=1}^{6} \omega_i [W_e(\lambda_i) + W_c(\lambda_i)]$<br>$f(J)$: controls the response to compression<br>$W_e(\lambda_i)$: strain energy of the elastin fiber<br>$W_c(\lambda_i)$: strain energy of the undulated bundle of collagen fibers<br>$\rho_0 W_c = E_c \left[\frac{1}{4(x_3 - x_1)}\left[(x_3 - x_1)(x_3 - 3x_1) + 2x_1^2 \ln\left(\frac{x_3}{x_1}\right)\right] + \left[\frac{x_3 - x_1}{2} - x_3\right] \ln\left(\frac{\lambda}{x_3}\right) + (\lambda - x_3)\right], \lambda > x_3$ (step function)<br>The orthotropic hyperelastic Langevin-based model with a strain energy function of the form [19]<br>$\rho_0 W = \rho_0 W_0 + \frac{nk\theta}{4}\left[N \sum_{i=1}^{4}\left(\frac{\rho^{(i)}}{N}\beta_\rho^{(i)} + \ln\frac{\beta_\rho^{(i)}}{\sinh\beta_\rho^{(i)}}\right) - \frac{\beta_P}{P}\ln\left(\lambda_a^{a^2}\lambda_b^{b^2}\lambda_c^{c^2}\right)\right] + B\{\cosh(J-1) - 1\}$<br>$W_0$: constant related to the strain energy of undeformed fibers<br>n: number of fibers per unit volume of the network<br>k: Boltzman's constant, $\sim 1.38 \times 10^{-23}$JK^{-1}<br>$\theta$: absolute temperature<br>$N$: number of freely jointed rigid links<br>$\beta_\rho^{(i)} = L^{-1}(\rho^{(i)}/N)$: Inverse Langevin function where $L(x) = \coth x - 1/x$<br>P : undeformed fiber length; $P = \sqrt{a^2 + b^2 + c^2}/2$, a, b, and c are the orthotropic cell dimensions

			$\left.\begin{matrix}\lambda_a = \sqrt{a^T C a}\\ \lambda_b = \sqrt{b^T C b}\\ \lambda_c = \sqrt{c^T C c}\end{matrix}\right\}$: principal stretches along the principal material axes for network. $\mathbf{C} = \mathbf{F}^{\mathbf{T}}\mathbf{F}$ is the right Cauchy stress tensor. $\boldsymbol{a}, \boldsymbol{b}$, and $\boldsymbol{c}$ from the axes that describe the orientation of the orthotropic cell<br>B: bulk modulus<br>J: volume ratio; $J = \det \mathbf{F}$
Bischoff orthotropic eight-chain	orthotropic, nonlinear, hyperelastic	rabbit abdominal skin [20], facial skin [6]	$W(x) = W_0 + \frac{nk\Theta}{4}\left(N \sum_{i=1}^{4}\left[\frac{\rho^{(i)}}{N}\beta_\rho^{(i)} + \ln\frac{\beta_\rho^{(i)}}{\sinh\beta_\rho^{(i)}}\right] - \frac{\beta_\rho}{\sqrt{N}}\ln\left[\lambda_a^{a^2}\lambda_b^{b^2}\lambda_c^{c^2}\right]\right) + \frac{B}{\alpha^2}\{\cosh[\alpha(J-1)] - 1\}$<br>$W_0$: constant representing zero-energy reference state<br>n: number density of macromolecular chains per unit volume<br>k: Boltzmann's constant<br>Θ: absolute temperature<br>N : number of rigid links in a chain (chain extensibility parameter)<br>$\rho^{(i)}$: deformed length of chain iii<br>$\beta_\rho^{(i)}$: inverse Langevin function evaluated at $\frac{\rho^{(i)}}{N}$<br>β_ρ: inverse Langevin function at reference chain length<br>$\lambda_a, \lambda_b, \lambda_c$: stretch ratios along principal material directions a, b, c<br>a, b, c : aspect ratios (dimensions) of the orthotropic unit cell<br>B : bulk modulus (controls near-incompressibility)<br>α: Curvature parameter in the bulk energy term (controls volume-change stiffness)<br>J: Determinant of deformation gradient det(F), represents volume ratio
Bischoff orthotropic eight-chain	orthotropic, nonlinear, hyperelastic	Human facial skin [6]	$W_{Bischoff} = \frac{nk\theta}{4}\left(p^2\left[\frac{p^{(i)}}{P^2}\beta_p^{(i)} + \ln\frac{\beta_p^{(i)}}{\sinh\beta_p^{(i)}}\right] - \frac{\beta_p}{P}\ln\left[\tilde{\lambda}_a^{a^2}\tilde{\lambda}_b^{b^2}\tilde{\lambda}_c^{c^2}\right]\right) + U(J)$ [6]<br>n: number of fibers per unit volume<br>k: Bolzman's constant<br>θ: absolute temperature

			Principal stretch<br>$\tilde{\lambda}_a^{a^2} = \boldsymbol{a^T}.\widetilde{\boldsymbol{C}}.\boldsymbol{a}$<br>$\tilde{\lambda}_b^{b^2} = \boldsymbol{b^T}.\widetilde{\boldsymbol{C}}.\boldsymbol{b}$<br>$\tilde{\lambda}_c^{c^2} = \boldsymbol{c^T}.\widetilde{\boldsymbol{C}}.\boldsymbol{c}$<br>$\boldsymbol{a},\boldsymbol{b},\boldsymbol{c}$**:** principal material axis<br>$\beta_p^{(i)} = \mathcal{L}^{-1}\left(\frac{p^{(i)}}{N}\right)$<br>$U(J) = \frac{B}{2}(\ln J)^2$, $B = 1MPa$ (bulk modulus)
Bischoff orthotropic eight-chain	orthotropic, nonlinear, viscoelastic	rabbit abdominal skin [21]	The strain energy density function for deviatoric model<br>$W = W_0 + \frac{n}{4}\sum_{i=1}^{4} w\left(\tilde{\rho}^{(i)}\right) - \frac{n}{4P}\left(\frac{dw(\tilde{\rho})}{d\tilde{\rho}}\right)_{\tilde{\rho}=P} \ln \tilde{\lambda}_a^{a^2}\tilde{\lambda}_b^{b^2}\tilde{\lambda}_c^{c^2} + \kappa[\cosh(J-1)-1]$<br>(Uncoupled [21])<br>$W_0$: strain energy density of the undeformed continuum<br>n: material parameter related to the fiber density<br>$\tilde{\rho}^{(i)} = \sqrt{\boldsymbol{P}^{(i)T}.\widetilde{\boldsymbol{C}}.\boldsymbol{P}^{(i)}}$: the deformed length of a constituent fiber in the unit cell<br>$\boldsymbol{P}^{(i)}$: vector description of fiber<br>$P = \sqrt{a^2+b^2+c^2}/2$: undeformed length of constitutive fiber<br>$w(\tilde{\rho})$: strain energy density of single fiber<br>$\tilde{\lambda}_a^{a^2}\tilde{\lambda}_b^{b^2}\tilde{\lambda}_c^{c^2}$: deviatoric stretches along the principal material directions (e.g $\tilde{\lambda}_a^{a^2} = \boldsymbol{a^T}.\widetilde{\boldsymbol{C}}.\boldsymbol{a}$)<br>$\kappa$: bulk modulus<br>The fiber strain energy density function is defined as<br>$w(\rho) = w_0 + k\Theta\mathrm{N}\left(\frac{\rho}{N}\beta + \ln\frac{\beta}{\sinh\beta}\right)$<br>$w_0$: strain energy density of an undeformed fiber<br>k: Boltzmann's constant<br>Θ: absolute temperature<br>N: the locking stretch of the chain and is related to the reference chain length according to the relation $\mathrm{N} = P^2$<br>$\beta = \mathcal{L}(\rho/N)$, $\mathcal{L}(x) = \coth(x) - 1/x$ (Langvin function)<br>Element A (stable elastin network), Element B (viscous fiber network):<br>n_A, n_B: chain density

Bischoff orthotropic eight-chain	orthotropic, nonlinear, QLV	Procine skin [22]	a_A, a_B: unit cell dimension along direction a<br>b_A, b_B: unit cell dimension along direction b<br>c_A, c_B: unit cell dimension along direction c<br>κ_A, κ_B: bulk modulus<br>Viscous (reptation) component:<br>$\dot{\gamma}_0$: base reptation rate (toggle for time dependence)<br>ϵ: regularisation constant (avoids numerical issues near zero strain)<br>$\boldsymbol{C}$**:** controls magnitude of viscous slip<br>$\boldsymbol{s_{base}}$**:** reference stress for scaling<br>$\boldsymbol{m}$**:** strain-rate sensitivity exponent<br><br>(Constrained, incompressible [22])<br>$W = \frac{n}{4}\left[\sum_{i=1}^{4} w\left(\lambda^{(i)}\right) - \frac{S_0}{2\Lambda} ln\left(\lambda_a^{a^2}\lambda_b^{b^2}\lambda_c^{c^2}\right)\right]$<br>The resulting continuum model<br>$\mathbf{S} = \frac{n}{8\Lambda}\sum_{i=1}^{4}\left[S\left(\lambda^{(i)}, t\right)\mathbf{P}^{(i)} \otimes \mathbf{P}^{(i)}\right] + \frac{nS_0^e}{8\Lambda}\left[a^2\left(1 - \frac{1}{\lambda_a^2}\right) \times a \otimes a + b^2\left(1 - \frac{1}{\lambda_b^2}\right) b \otimes b + c^2\left(1 - \frac{1}{\lambda_c^2}\right) c \otimes c\right]$<br>$w\left(\lambda^{(i)}\right)$: the strain energy function for a constituent fiber class as a function of its stretch $\lambda^{(i)}$<br>n: material parameter that reflects fiber density<br>a, b, and c: material parameters that reflect the unit cell dimensions<br>τ_1, τ_2: short/long relaxation time<br>S_0 : 2nd Piola–Kirchhoff (PK) stress of the undeformed fiber<br>λ_a, λ_b, and λ_c: stretches along the three material directions<br>$\Lambda = (a^2 + b^2 + c^2)/8$: fiber contour length<br>$c$: damping parameter

References

[1] O. A. Shergold, N. A. Fleck, and D. Radford, “The uniaxial stress versus strain response of pig skin and silicone rubber at low and high strain rates,” *Int J Impact Eng*, vol. 32, no. 9, pp. 1384–1402, Sep. 2006, doi: 10.1016/j.ijimpeng.2004.11.010.

[2] C. Bellini, P. Glass, M. Sitti, and E. S. Di Martino, “Biaxial mechanical modeling of the small intestine,” *J Mech Behav Biomed Mater*, vol. 4, no. 8, pp. 1727–1740, Nov. 2011, doi: 10.1016/j.jmbbm.2011.05.030.

[3] J. C. Simo and R. L. Taylor, “Quasi-incompressible finite elasticity in principal stretches. continuum basis and numerical algorithms,” *Comput Methods Appl Mech Eng*, vol. 85, no. 3, pp. 273–310, 1991, doi: 10.1016/0045-7825(91)90100-K.

[4] C. Flynn, A. Taberner, and P. Nielsen, “Modeling the Mechanical Response of In Vivo Human Skin Under a Rich Set of Deformations,” *Ann Biomed Eng*, vol. 39, no. 7, pp. 1935–1946, Jul. 2011, doi: 10.1007/s10439-011-0292-7.

[5] P. Tong and Y. C. Fung, “The stress-strain relationship for the skin,” *J Biomech*, vol. 9, no. 10, pp. 649–657, Jan. 1976, doi: 10.1016/0021-9290(76)90107-X.

[6] C. Flynn, A. T. Taberner, S. Fels, and P. M. F. Nielsen, “Comparison of Anisotropic Models to Simulate the Mechanical Response of Facial Skin,” in *Lecture Notes in Bioengineering*, 2018, pp. 43–55. doi: 10.1007/978-3-319-59764-5_6.

[7] G. A. Ateshian and K. D. Costa, “A frame-invariant formulation of Fung elasticity,” *J Biomech*, vol. 42, no. 6, pp. 781–785, Apr. 2009, doi: 10.1016/J.JBIOMECH.2009.01.015,.

[8] Y. C. Fung, K. Fronek, and P. Patitucci, “Pseudoelasticity of arteries and the choice of its mathematical expression,” *Am J Physiol Heart Circ Physiol*, vol. 6, no. 5, 1979, doi: 10.1152/AJPHEART.1979.237.5.H620,.

[9] G. A. Holzapfel, T. C. Gasser, and R. W. Ogden, “Comparison of a Multi-Layer Structural Model for Arterial Walls With a Fung-Type Model, and Issues of Material Stability,” *J Biomech Eng*, vol. 126, no. 2, pp. 264–275, Apr. 2004, doi: 10.1115/1.1695572.

[10] A. Ní Annaidh *et al.*, “Automated Estimation of Collagen Fibre Dispersion in the Dermis and its Contribution to the Anisotropic Behaviour of Skin,” *Ann Biomed Eng*, vol. 40, no. 8, pp. 1666–1678, Aug. 2012, doi: 10.1007/s10439-012-0542-3.

[11] A. Aldieri, M. Terzini, C. Bignardi, E. M. Zanetti, and A. L. Audenino, “Implementation and validation of constitutive relations for human dermis mechanical response,” *Med Biol Eng Comput*, vol. 56, no. 11, pp. 2083–2093, Nov. 2018, doi: 10.1007/S11517-018-1843-Y/FIGURES/6.

[12] W. Li and X. Y. Luo, “An Invariant-Based Damage Model for Human and Animal Skins,” *Ann Biomed Eng*, vol. 44, no. 10, pp. 3109–3122, Oct. 2016, doi: 10.1007/S10439-016-1603-9/FIGURES/8.

[13] T. C. Gasser, R. W. Ogden, and G. A. Holzapfel, “Hyperelastic modelling of arterial layers with distributed collagen fibre orientations,” *J R Soc Interface*, vol. 3, no. 6, pp. 15–35, Feb. 2006, doi: 10.1098/rsif.2005.0073.

[14] J. W. Y. Jor, M. P. Nash, P. M. F. Nielsen, and P. J. Hunter, “Estimating material parameters of a structurally based constitutive relation for skin mechanics,” *Biomech Model Mechanobiol*, vol. 10, no. 5, pp. 767–778, Nov. 2011, doi: 10.1007/S10237-010-0272-0/METRICS.

[15] R. B. Groves, S. A. Coulman, J. C. Birchall, and S. L. Evans, “An anisotropic, hyperelastic model for skin: Experimental measurements, finite element modelling and identification of parameters for human and murine skin,” *J Mech Behav Biomed Mater*, vol. 18, pp. 167–180, Feb. 2013, doi: 10.1016/j.jmbbm.2012.10.021.

[16] J. A. Weiss, B. N. Maker, and S. Govindjee, “Finite element implementation of incompressible, transversely isotropic hyperelasticity,” *Comput Methods Appl Mech Eng*, vol. 135, no. 1–2, pp. 107–128, 1996.

[17] J. M. Vassoler, L. Reips, and E. Fancello, “A variational framework for fiber-reinforced viscoelastic soft tissues,” *Int J Numer Methods Eng*, vol. 89, no. 13, pp. 1691–1706, 2012.

[18] M. B. Rubin and S. R. Bodner, “A three-dimensional nonlinear model for dissipative response of soft tissue,” *Int J Solids Struct*, vol. 39, no. 19, pp. 5081–5099, Sep. 2002, doi: 10.1016/S0020-7683(02)00237-8.

[19] C. Flynn, M. B. Rubin, and P. Nielsen, “A model for the anisotropic response of fibrous soft tissues using six discrete fibre bundles,” *Int J Numer Method Biomed Eng*, vol. 27, no. 11, pp. 1793–1811, 2011.

[20] J. E. Bischoff, E. A. Arruda, and K. Grosh, “A microstructurally based orthotropic hyperelastic constitutive law,” *J. Appl. Mech.*, vol. 69, no. 5, pp. 570–579, 2002.

[21] J. E. Bischoff, E. M. Arruda, and K. Grosh, “A rheological network model for the continuum anisotropic and viscoelastic behavior of soft tissue,” *Biomech Model Mechanobiol*, vol. 3, no. 1, pp. 56–65, Sep. 2004, doi: 10.1007/s10237-004-0049-4.

[22] J. E. Bischoff, “Reduced parameter formulation for incorporating fiber level viscoelasticity into tissue level biomechanical models,” *Ann Biomed Eng*, vol. 34, pp. 1164–1172, 2006.
